# Anti-GPV activity of *Lactobacillus*-fermented traditional Chinese medicines

**DOI:** 10.3389/fmicb.2026.1738123

**Published:** 2026-02-10

**Authors:** Hongrui Chen, Songrui Liu, Qiuxuan Wang, Hanjia Zhang, Meng Qingfeng, Hao Dong

**Affiliations:** 1 College of Life Sciences, Jilin Agricultural University, Changchun, Jilin, China; 2 Ministry of Education Engineering Research Center for Bioreactors and Drug Development, College of Life Sciences, Jilin Agricultural University, Changchun, Jilin, China

**Keywords:** antiviral activity, bioconversion, fermented Chinese medicinal materials, GPV, metabolites, microbial fermentation, network, pharmacology

## Abstract

**Background:**

Honeysuckle and gardenia are classified as cold-natured traditional Chinese medicinal herbs, with chlorogenic acid and gardenoside recognized as their major bioactive components. Modern pharmacological studies have confirmed their antiviral, anti-inflammatory, antioxidant, and immunomodulatory activities. However, research on the microbial fermentation of traditional Chinese medicine (TCM) for antiviral applications remains limited. This study aimed to investigate the antiviral activity against Goose parvovirus (GPV) of fermented TCM using *Lactobacillus* isolated from silage feed, and to explore its potential theoretical and practical value.

**Methodology:**

*Lactobacillus plantarum* was isolated and identified using standard microbiological methods, and its probiotic properties were evaluated. The *in vitro* antiviral activity of bacterial cells and their metabolites was assessed using cellular models, including the cell counting kit-8 (CCK-8) assay and quantitative PCR (qPCR). High-performance liquid chromatography (HPLC) was employed to analyze changes in active components of TCM following lactic acid fermentation. In addition, network pharmacology and molecular docking analyses were performed to elucidate the potential mechanisms of action.

**Results:**

A strain of *Lactobacillus plantarum* with strong acid and bile salt tolerance (survival rate > 92%) was successfully isolated. The strain effectively inhibited pathogenic bacteria and demonstrated safety in mice. *In vitro* experiments showed that bacterial metabolites significantly suppressed GPV proliferation. Fermentation markedly increased the contents of active components, including chlorogenic acid and gardenoside, in honeysuckle and gardenia decoctions. Moreover, the fermented mixed decoction exhibited a highly significant anti-GPV effect. Network pharmacology and molecular docking analyses indicated that key active components, such as quercetin, exert antiviral, and anti-inflammatory effects mainly through the regulation of Toll-like receptor–related signaling pathways involving targets such as IL-6 and TNF.

**Conclusion:**

This study demonstrates that *L. plantarum*, TCM, and their fermentation products effectively alleviate GPV infection and improve intestinal barrier function. HPLC and network pharmacology analyses suggest that fermentation-derived active components, including chlorogenic acid, gardenoside, quercetin, and organic acids, may synergistically enhance antiviral and anti-inflammatory effects by modulating IL-6/TNF-related signaling pathways and interacting with the gut microbiota.

## 1 Research background

Piglet plague is an acute septicemic infectious disease caused by Goose parvovirus (GPV), primarily affecting goslings and ducklings under 3 weeks of age (Lu et al., 2025). The disease is characterized by high contagiousness and mortality, resulting in substantial economic losses to the goose farming industry (Siedlecka et al., 2025). Recent epidemiological investigations have shown an expansion in the age range of affected birds, with sporadic outbreaks occurring irregularly, which has increased the difficulty of effective disease control and attracted widespread attention (Zhu et al., 2025). GPV exhibits strong environmental stability, maintaining infectivity after exposure to pH 3.0 for 1 h and to 56 °C for 3 h (Makhija and Kumar, 2017). The virus has a single serotype and does not exhibit cross-serological reactions with other viruses within the same genus; however, it shares partial common antigens with Muscovy duck parvovirus. Unlike most parvoviruses, GPV is capable of autonomous replication, allowing viral proliferation independent of host cell mitosis (Lopez-Astacio et al., 2023). *In vitro* studies have demonstrated that certain GPV strains can successfully replicate in goose embryo fibroblasts, Muscovy duck embryo fibroblasts, and duck embryo fibroblasts (Nayga et al., 2025).

Traditional Chinese medicine (TCM), an integral component of China’s traditional medical heritage, has attracted increasing attention from both domestic and international research communities. This growing interest is largely attributed to its abundant natural resources, diverse bioactive components, and relatively low potential for drug resistance (Li et al., 2022). The application of TCM has a long history, evolving from ancient distillation techniques to modern steam extraction of essential oils, reflecting continuous exploration and utilization of its active constituents. In recent years, fermentation technology has been introduced into the processing of Chinese herbal medicines as a simple, low-cost, and efficient bioconversion approach, leading to the development of fermentation-based techniques for herbal medicine production (Chen et al., 2022). This process utilizes microorganisms to catalyze the transformation and degradation of herbal components under appropriate conditions, thereby enhancing therapeutic efficacy, generating novel bioactive compounds, and simultaneously reducing toxicity and adverse effects. Modern fermentation methodologies have further improved product quality, stability, and reproducibility through the adoption of standardized production procedures. With increasing concerns regarding antibiotic resistance, environmental pollution, and product safety resulting from the excessive use of antibiotics in animal husbandry, fermented Chinese medicinal materials have emerged as low-residue and low-toxicity alternatives, becoming a research hotspot in the field of feed additives (Cãtanã et al., 2018). Studies have demonstrated that microbial fermentation can release active components entrapped within plant cell walls through enzymatic hydrolysis—for example, increasing the total saponin content of fermented *Astragalus membranaceus* by approximately 22% (Silva et al., 2019). In addition, fermentation can convert herbal constituents into novel bioactive substances, such as the production of 6-O-β-D-glucosyl-cis-astragaloside through *Ganoderma lucidum* fermentation of *Astragalus* (Ruan et al., 2010), and reduce herbal toxicity via microbial metabolism, as observed in fermented *Strychnos nux-vomica*, which exhibits significantly lower toxicity while maintaining therapeutic efficacy (Almeida et al., 2020). Moreover, the application of fermented Chinese herbs as feed additives has been shown to significantly improve animal growth performance, with reported reductions in feed conversion ratio (FCR) of up to 24.85%. A concomitant decrease in diarrhea incidence has also been observed, indicating broad potential for enhancing immune function and feed utilization efficiency in animal production systems (Chen et al., 2023).

The present study aims to explore a novel strategy for the prevention and control of GPV by employing lactic acid bacteria isolated from silage feed to ferment Chinese herbal medicines. Chinese herbal medicines have demonstrated distinct advantages in antiviral activity (Cheng et al., 2021), while lactic acid bacteria are known to enhance therapeutic efficacy by regulating gut microbiota, improving immune function, and degrading macromolecular herbal constituents (Bergervoet et al., 2019). However, systematic investigations into the combined application of these approaches remain limited (Caiazzo et al., 2022). In this study, lactic acid bacteria were isolated and screened, their probiotic properties were evaluated, and selected strains were applied to ferment honeysuckle and gardenia. Multiple analytical approaches were used to characterize compositional changes and antiviral activity before and after fermentation, including *in vitro* assays and network pharmacology analysis. In addition, the safety and potential mechanisms of action were assessed. The results of this study provide both a theoretical basis and practical support for the development of novel biopreparations as alternatives to antibiotics for GPV control. This work addresses an existing gap in the application of fermented Chinese herbal medicines for the prevention and control of avian viral diseases (He et al., 2021).

## 2 Materials and methods

### 2.1 Drugs and strains

All herbal materials, including honeysuckle and gardenia, were purchased from Jilin Dayao Pharmacy Co., Ltd. Silage samples were provided by the Straw Fermentation Platform located on the third floor of the Science and Technology Innovation Building, College of Life Sciences, Jilin Agricultural University.

The compound herbal preparation was formulated in accordance with the *Pharmacopoeia of the People’s Republic of China*. Briefly, honeysuckle and gardenia (20 g each) were pulverized to 800 mesh at room temperature. The powdered materials were mixed with 1,000 mL of distilled water in a decoction pot, soaked for 2 h, and then decocted for 55 min. The residue was subsequently decocted with an additional 500 mL of distilled water and filtered through double-layer gauze. This second decoction was maintained for 45 min. The decoction process was repeated twice, with double-layer gauze filtration applied after each cycle. The combined filtrates from all three decoctions were concentrated to approximately 10 mL, sterilized by autoclaving, and stored at 4°C for subsequent use.

For bacterial isolation, 5 g of fermented silage was weighed, cut into small pieces, and suspended in 100 mL of de Man–Rogosa–Sharpe (MRS) liquid medium. The suspension was incubated in a shaking incubator at 37°C for at least 48 h. Subsequently, 100 μL of the culture was spread onto sterilized MRS agar plates supplemented with 2% CaCO3. After anaerobic incubation at 37°C for 48 h, presumptive colonies were randomly selected and repeatedly streaked on MRS agar plates for purification. A single purified *Lactobacillus* colony was inoculated into MRS liquid medium and cultured at 37°C under sealed conditions for 36 h. The concentration of the freshly cultured bacterial suspension was adjusted to 1.0 × 10^8^ CFU/mL. An inoculum volume equivalent to 4% (v/v) was added to the prepared Chinese herbal decoction, which was then fermented at 37°C under sealed conditions for 36 h.

### 2.2 Animals and experimental design

Sixteen healthy, 3-week-old male specific pathogen-free (SPF) Kunming mice, weighing approximately 20 g, were obtained from the Laboratory Animal Center of Jilin University (Changchun, Jilin Province, China). The mouse model was used exclusively to evaluate the *in vivo* biosafety and probiotic characteristics of the isolated *Lactobacillus plantarum* strain and was not intended to assess antiviral efficacy against Goose parvovirus. All animals were housed under SPF conditions with free access to standard chow and water. Environmental conditions were strictly controlled at a temperature of 22–25°C with a 12-h light/dark cycle. All experimental procedures were approved by the Laboratory Animal Ethics Committee of Jilin Agricultural University (Changchun, Jilin, China) and were conducted in accordance with the institutional guidelines for animal care and use.

Following a 2-week acclimatization period, the mice were randomly divided into two groups (*n* = 8 per group), designated as Group A and Group B. Mice in Group B received a daily oral gavage of 1 mL bacterial suspension at a concentration of 1.0 × 10^8^ CFU/mL, while Group A served as the control and was administered an equal volume of physiological saline. During the 2-week experimental period, all mice had *ad libitum* access to food and water. Feed intake, general behavior, and health status were monitored daily throughout the experiment. On day 15, all surviving mice were euthanized. To ensure tissue integrity and adherence to animal welfare regulations, cervical dislocation was performed by trained personnel, followed by confirmation of death. This euthanasia method was approved by the Laboratory Animal Ethics Committee of Jilin Agricultural University. Intestinal tissues were collected for autopsy and histological analysis, and spleen tissues were examined microscopically after hematoxylin and eosin (HE) staining.

### 2.3 Cells and viruses

Goose embryonic fibroblasts (GEFs) were prepared from primary cultures in strict accordance with established laboratory protocols. Briefly, embryos at 12 days post-fertilization were dissected under sterile conditions. After repeated washing with phosphate-buffered saline (PBS), the head, limbs, and visceral tissues were removed. The remaining tissues were minced and digested with 0.25% trypsin at 37°C for 9 min. The digestion was terminated, and the cells were resuspended in Dulbecco’s modified Eagle’s medium (DMEM). The cell suspension was filtered and seeded into culture dishes. GEFs were maintained in complete DMEM supplemented with serum at 37°C in a humidified incubator containing 5% CO_2_. Cell attachment and growth were examined the following day microscopically.

The GPV strain used in this study was propagated in our laboratory and verified to meet experimental requirements for purity and virulence. For viral propagation, GPV was inoculated onto confluent GEF monolayers at an appropriate multiplicity of infection (MOI). After viral adsorption, the cells were cultured in fresh medium and monitored periodically for cytopathic effects (CPE). When CPE exceeded 80%, the culture supernatant was harvested and centrifuged to remove cellular debris. The clarified virus stock was aliquoted and stored at -80°C for subsequent experiments.

### 2.4 DNA extraction, 16S rRNA analysis, and growth curve determination

Total bacterial genomic DNA was extracted using a rapid bacterial genomic DNA extraction kit, following the manufacturer’s instructions. The target 16S rRNA gene fragments were amplified using specific primers, and the PCR products were verified by agarose gel electrophoresis. The amplified products were subsequently sequenced by Sangon Biotech (Shanghai) Co., Ltd. The obtained sequences were compared with those in the GenBank database using the BLAST program, and a phylogenetic tree was constructed using MEGA version 7.0 software based on the neighbor-joining method. To assess bacterial growth characteristics, single colonies were inoculated into MRS broth and incubated overnight at 37°C. The cultures were then serially diluted into fresh MRS medium for further cultivation. Incubation was carried out at 37°C for 36 h, during which samples were collected at 2-h intervals to measure optical density at 600 nm (OD_600_) and pH values. Growth curves and acid production curves were subsequently generated.

### 2.5 Acid and bile salt tolerance assays, antimicrobial activity, and antibiotic sensitivity testing

To evaluate the probiotic properties of the isolated *Lactobacillus* strains, a series of assays was performed, including acid tolerance, bile salt tolerance, antimicrobial activity, and antibiotic susceptibility tests. For acid tolerance assessment, 1 mL of bacterial suspension was inoculated into 9 mL of MRS broth adjusted to pH 2.5. Following anaerobic incubation at 37°C for 3 h, viable cell counts before and after incubation were determined using the plate count method. For bile salt tolerance analysis, 1 mL of bacterial suspension was inoculated into MRS broth supplemented with 0.3% (w/v) ox bile salts and incubated under identical conditions for 8 h. Cell viability was assessed using the same plate count procedure. Antimicrobial activity was evaluated using the agar diffusion method. Indicator strains (10^7^ CFU/mL) were evenly spread onto Luria-Bertani (LB) agar plates. Wells were then punched into the agar, and *Lactobacillus* suspensions were added to the wells, with MRS broth serving as the negative control. After incubation at 37°C for 48 h, the diameters of the inhibition zones were measured to assess antimicrobial activity. Antibiotic susceptibility testing was performed using the Kirby-Bauer disk diffusion method. Bacterial suspensions were adjusted to a concentration of 10^5^ CFU/mL and spread onto MRS agar plates. Antibiotic discs were spread on the plates, followed by incubation at 37°C for 24–48 h. The diameters of inhibition zones were measured to determine susceptibility to antibiotics, including penicillin.

### 2.6 Determination of GPV titers and growth curves in infected GEFs

GEFs were seeded into 96-well plates and cultured until cell attachment was achieved. The GPV stock was serially diluted tenfold to 10^−10^, and each dilution was inoculated into eight replicate wells, with uninfected cells serving as the cell control. After incubation at 37°C for 5 days, CPE were observed. Viral titers were calculated using the Kärber method and expressed as 50% tissue culture infective dose (TCID_50_/100 μL). For viral growth curve analysis, GEFs were seeded into 12-well plates and cultured to approximately 90% confluence. The GPV suspension, pre-adsorbed with trypsin for 1.5 h, was inoculated onto the cell monolayers. After viral adsorption, the cells were washed with PBS and cultured in fresh DMEM. Cell samples were collected at 12, 24, 36, 48, and 60 h post-infection. Total RNA was extracted and reverse-transcribed into cDNA. Quantitative real-time PCR was performed to determine cycle threshold (Ct) values, which were used to analyze GPV replication dynamics.

### 2.7 Determination of maximum safe dose of lactobacillus and its metabolites and evaluation of antiviral activity against GPV

To assess the antiviral effects of *Lactobacillus* and its metabolites against GPV, a GEF infection model was established with different treatment strategies. GEFs were seeded into 96-well plates and cultured to 80–90% confluence. *Lactobacillus* suspensions, metabolic extracts, and heat-inactivated products were serially diluted and added to the designated wells to determine the maximum safe concentration. After incubation for 1.5 h at 37°C in a 5% CO_2_ atmosphere, the medium was replaced with maintenance medium. The experiment included three treatment groups: Group A (pretreatment group), in which *Lactobacillus* preparations were administered prior to viral infection; Group B (co-treatment group), in which *Lactobacillus* preparations were mixed with GPV and added simultaneously; and Group C (post-treatment group), in which cells were first infected with GPV, followed by *Lactobacillus* administration. Cell control and virus control groups were included in all experiments. GPV infection was performed using a 100 TCID_50_ virus suspension for 1.5 h. After 36 h of incubation, cell counting kit-8 (CCK-8) reagent was added to each well and incubated for an additional 2 h. Absorbance at 450 nm was measured using a microplate reader to evaluate cell viability and antiviral activity.

### 2.8 Quantitative real-time PCR analysis of the anti-GPV activity of lactobacillus suspensions and their metabolites

Under aseptic conditions, 2 mL of GEF suspension was seeded into 6-well plates. After incubation for 12 h to allow cell attachment, the cells were washed three times with PBS. The maximum non-cytotoxic concentrations of *Lactobacillus* suspension and its metabolites were each mixed with an equal volume of GPV suspension (100 TCID50). Following co-incubation for 1.5 h at 37°C, the inoculum was removed and replaced with maintenance medium. A cell control group, consisting of cells cultured in maintenance medium only, and a virus control group, consisting of cells infected with GPV mixed with maintenance medium, were established and maintained under identical conditions. After 36 h of incubation, the plates were subjected to three freeze-thaw cycles between -80 and 4°C, followed by thorough pipetting to release viral particles. Viral RNA was then extracted and reverse-transcribed into cDNA. Viral copy numbers were quantified using a previously established quantitative real-time PCR (qPCR) assay.

### 2.9 High-performance liquid chromatography analysis of active component changes in fermented Chinese herbal decoctions

HPLC was employed to determine changes in the concentrations of chlorogenic acid and gardenoside in honeysuckle and gardenia decoctions before and after fermentation. For chlorogenic acid analysis, the mobile phase consisted of acetonitrile and 0.4% phosphoric acid aqueous solution (13:87, v/v), with detection performed at a wavelength of 327 nm. Test samples included honeysuckle extracts and corresponding *Lactobacillus*-fermented solutions diluted 500-fold with 50% methanol. The injection volume was 20 μL, the flow rate was set at 0.1 mL/min, and peak areas were recorded. Gardenoside analysis was performed using an acetonitrile-water mobile phase (15:85, v/v) with detection at 238 nm. A gardenoside reference standard was prepared by dissolving the compound in methanol to a concentration of 30 μg/mL. Honeysuckle extracts and fermentation broths were diluted 100-fold with methanol and analyzed under the same chromatographic conditions. In a separate experiment, 200 mL of a mixed honeysuckle and gardenia decoction was supplemented with 4.8% MRS broth and inoculated with 5% *Lactobacillus* suspension. Fermentation was carried out at 41.5°C for 42 h. Viable bacterial counts were determined at 6-h intervals during fermentation. Samples were collected at 24, 30, and 36 h, and changes in the concentrations of chlorogenic acid and other active components were analyzed under the chromatographic conditions described above. This approach enabled the evaluation of the dynamic variation of active compounds during the fermentation process.

### 2.10 Antiviral Activity of lactobacillus-fermented Chinese herbal decoctions against GPV

A series of experiments was conducted to evaluate the antiviral activity of different Chinese herbal decoctions and their corresponding fermented products against GPV. First, the maximum non-cytotoxic concentration was determined using the CCK-8 assay. GEFs were co-incubated with samples at concentrations ranging from 2 to 32 mg/mL for 1.5 h. After washing and replacement with fresh medium, the cells were further cultured for 48 h. Following the CCK-8 reaction, absorbance at 450 nm (OD_450_) was measured to calculate cell viability and determine the maximum safe concentration. Subsequently, antiviral efficacy was assessed using qPCR. GEFs were seeded into 6-well plates, and samples at the maximum non-cytotoxic concentration (MDTC) were mixed with an equal volume of GPV suspension (100 TCID_50_) and added to the cells. After 1.5 h of incubation, the inoculum was replaced with maintenance medium. After 36 h, viral supernatants were collected through repeated freeze-thaw cycles. Viral RNA was extracted, reverse-transcribed into cDNA, and viral copy numbers were quantified by qPCR. Finally, three treatment strategies, pretreatment, co-treatment, and post-treatment, were established to compare antiviral effects under different administration modes. In each group, GEFs were treated with samples at a concentration of 4 mg/mL either before, during, or after GPV infection, respectively, while the virus control group was infected simultaneously. After 36 h of continuous incubation, cell viability was evaluated using the CCK-8 assay to assess the antiviral activity of the different treatment strategies.

### 2.11 Network pharmacology and molecular docking analysis of the mechanisms of TCMs against GPV

Network pharmacology approaches were employed to explore the potential mechanisms of action of honeysuckle and gardenia against GPV infection. First, the active components of honeysuckle and gardenia were screened from the Traditional Chinese Medicine Systems Pharmacology (TCMSP) database based on the criteria of oral bioavailability (OB ≥ 30%) and drug-likeness (DL ≥ 0.18). Corresponding target genes were retrieved, and gene names were standardized using the UniProt database. Simultaneously, GPV-related targets were collected from the GeneCards, Online Mendelian Inheritance in Man (OMIM), and NCBI Gene databases. After integration and removal of duplicate entries, the intersecting targets between the active compound targets and disease-related targets were identified as potential therapeutic targets. Cytoscape software was used to construct a compound-target-disease interaction network. In parallel, the STRING database was utilized to generate a protein-protein interaction (PPI) network for screening core targets. Gene Ontology (GO) functional enrichment analysis and Kyoto Encyclopedia of Genes and Genomes (KEGG) pathway enrichment analysis were subsequently performed on the intersecting targets using the DAVID database to elucidate their biological functions and signaling pathways. Finally, molecular docking analysis was conducted to validate the binding affinity between key active components and core target proteins. Docking simulations and visualization were performed using AutoDock software.

### 2.12 Statistical analysis

All experimental data are presented as mean ± standard error of the mean (SEM). Statistical analyses were performed using GraphPad Prism version 6.0 (GraphPad Software Inc., San Diego, CA, United States). Differences between groups were evaluated using one-way analysis of variance (ANOVA) followed by Tukey’s multiple comparison test. A value of *P* < 0.05 was considered statistically significant.

## 3 Results

### 3.1 Molecular identification of the isolated strain by 16S rRNA analysis

**FIGURE 1:**
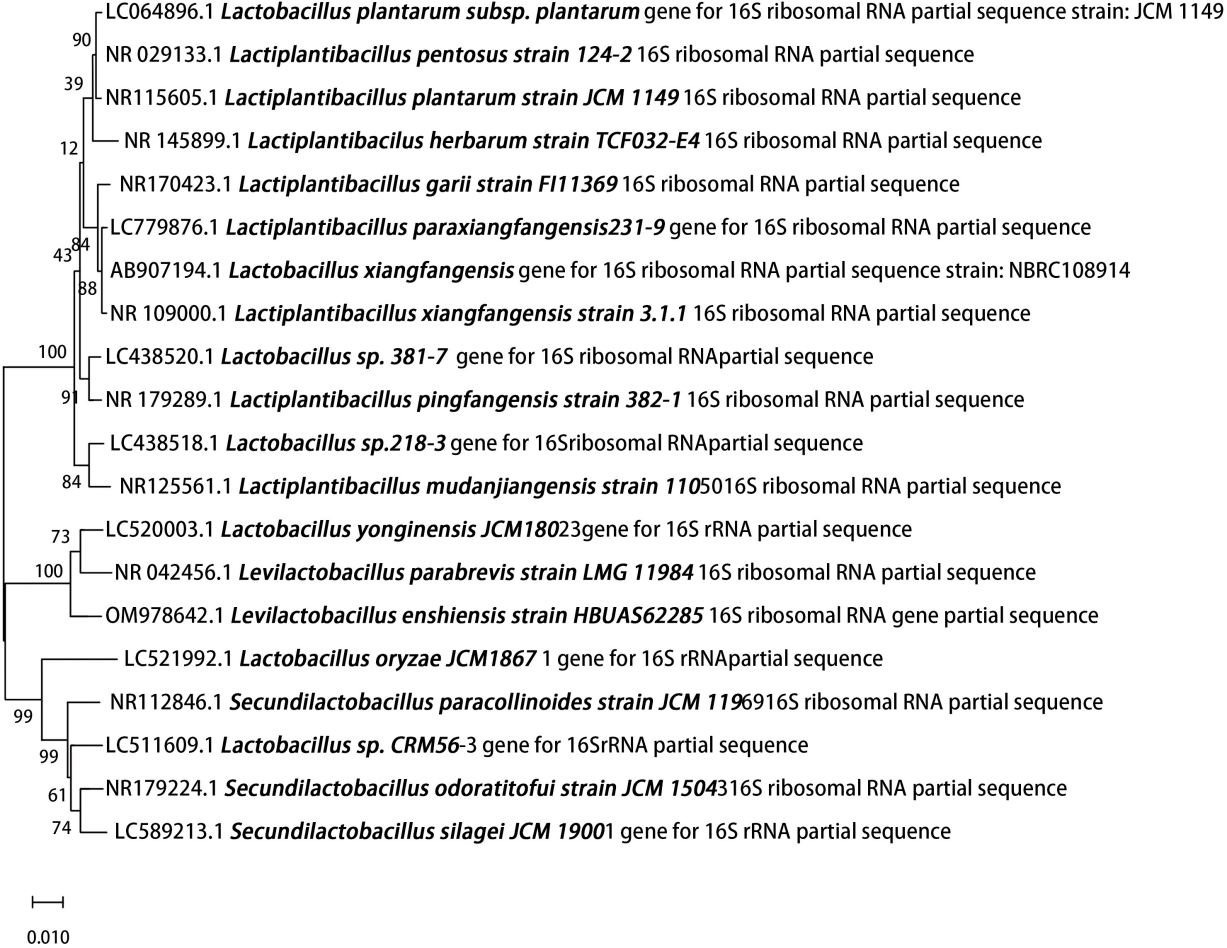
The 16S rRNA gene sequence of the isolated strain was compared with sequences in the GenBank database using the BLAST algorithm. A phylogenetic tree was constructed using the neighbor-joining method with MEGA 7.0 software, demonstrating the highest homology with *Lactobacillus plantarum*.

The isolated strain was conclusively identified as *Lactobacillus plantarum* based on 16S rRNA gene analysis. PCR amplification generated a single, clear band of approximately 1,500 bp, consistent with the expected fragment size (Supplementary Figure S1), indicating successful and specific amplification. Subsequent sequence alignment and homology analysis against the GenBank database revealed a high degree of sequence similarity to *L. plantarum*. Phylogenetic analysis further demonstrated that the isolated strain clustered tightly with reference *L. plantarum* strains, clearly distinguishing it from other Lactobacillus species (Figure 1), thereby confirming its taxonomic identity.

**FIGURE 2:**
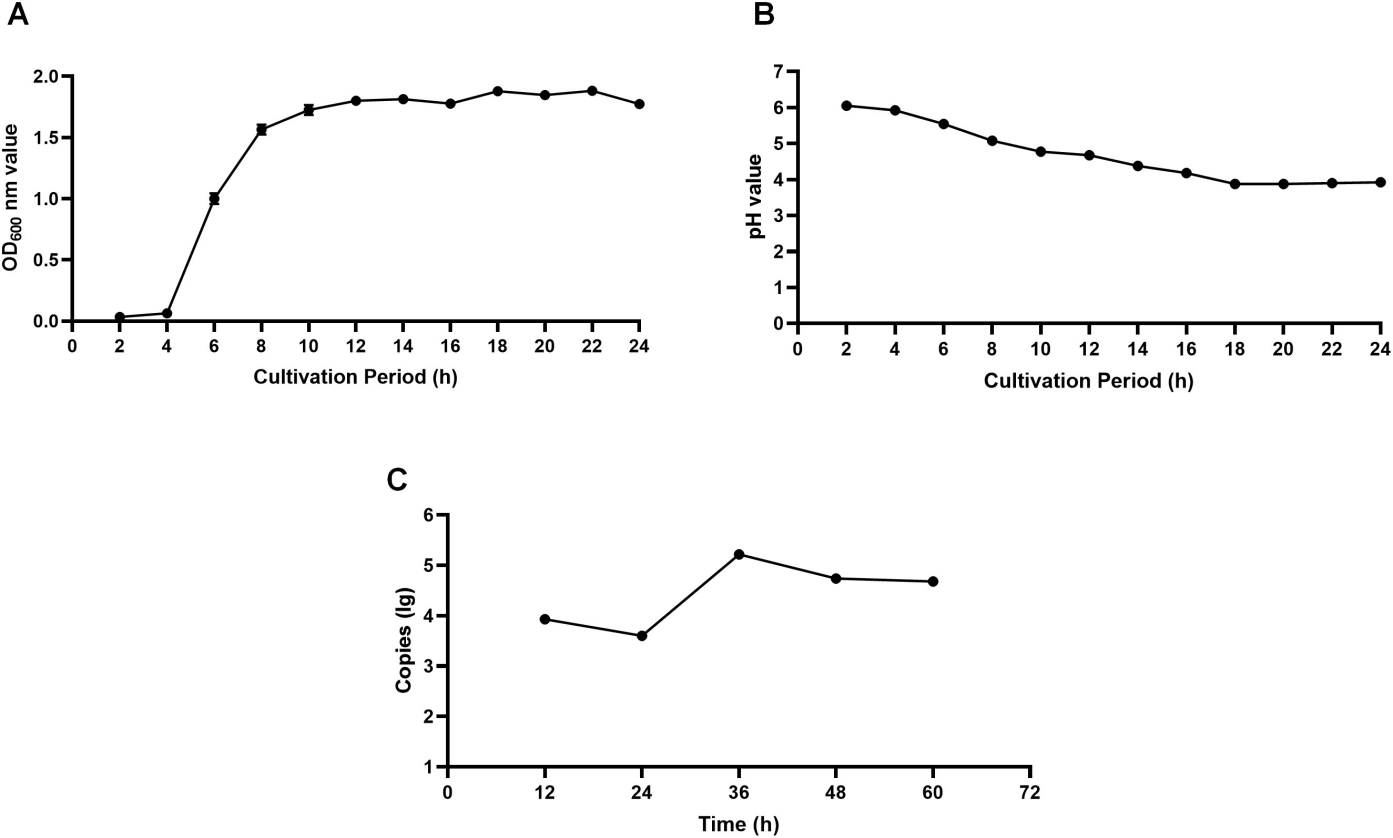
**(A)** The strain was inoculated into MRS broth and incubated at 37°C. The optical density at 600 nm (OD600) was measured every 2 h to monitor bacterial growth over a 36-h period. Data points represent the mean values of three independent experiments. **(B)** The pH of the bacterial culture supernatant was measured every 2 h during a 36-h incubation period at 37°C. The curve shows a rapid decline in pH during the logarithmic growth phase, followed by stabilization at a low pH, indicating strong acid-producing capacity. **(C)** GEFs were infected with GPV, and cell lysates were collected at the indicated time points (12, 24, 36, 48, 60 h). Viral RNA copies were quantified by qPCR. The viral load peaked at 36 h post-infection (hpi), reaching 10^5.22^ copies.

As shown in Figure 2A, the growth curve analysis indicated that the isolated *Lactobacillus* strain underwent a lag phase of approximately 4 h, followed by a rapid logarithmic growth phase, and subsequently entered the stationary phase after 18 h of incubation at 37°C, when the OD_600_ value reached its maximum. This growth pattern reflects typical proliferation dynamics of lactic acid bacteria under nutrient-sufficient conditions, and therefore 18 h was selected as the optimal harvest time to ensure high cell viability and metabolic activity. As shown in Figure 2B, the pH of the fermentation broth decreased markedly during the logarithmic growth phase (4–18 h), reaching a minimum value of 3.82, and then remained relatively stable thereafter. This rapid acidification is consistent with active carbohydrate metabolism and organic acid accumulation accompanying bacterial proliferation. The stabilization of pH during the stationary phase suggests a balance between acid production and metabolic slowdown, indicating that the strain possesses strong and sustained acid-producing capacity during active growth.

### 3.2 Acid and bile salt tolerance, antimicrobial activity, and antibiotic susceptibility of the isolated lactobacillus strains

As shown in Supplementary Table S1, after exposure to pH 2.5 for 3 h, the survival rates of all three *Lactobacillus* strains exceeded 97%. Following incubation in MRS medium containing 0.3% bile salts for 8 h, survival rates remained above 92%. Previous studies have indicated that a minimum viable count of 1.0 × 10^6^ CFU/mL is required for *Lactobacillus* to exert probiotic effects. Although the viable cell counts (log CFU/mL) of the isolated strains decreased under acidic and bile salt conditions, they remained above 1.0 × 10^7^ CFU/mL, which is substantially higher than the minimum effective threshold.

The antimicrobial activity of the isolated *Lactobacillus* strains against pathogenic bacteria was evaluated using the agar well diffusion method. As shown in Table 1, *Salmonella Typhimurium* exhibited high susceptibility to the strains, with a mean inhibition zone diameter of 17.00 ± 0.16 mm, while *Staphylococcus aureus* also showed strong susceptibility, with an inhibition zone diameter of 16.03 ± 0.21 mm. In contrast, enteropathogenic *Escherichia coli* demonstrated relatively low susceptibility, as reflected by a markedly smaller inhibition zone (7.80 ± 0.33 mm). These results indicate differential antibacterial activity of the isolated *Lactobacillus* strains against the tested pathogens, with stronger inhibitory effects against *S. Typhimurium* and *S. aureus* than against enteropathogenic *E. coli*.

**TABLE 1:** Bacteriostatic capacity of *Lactobacillus* isolates.

Strain	Antibacterial zone (mm)		
	Salmonella *Typhimurium*	Staphylococcus aureus	Enteropathogenic *Escherichia coli*
*Bacillus plantarum*	17.00 ± 0.16	16.03 ± 0.21	7.80 ± 0.33

The antibiotic susceptibility profiles of the isolated *Lactobacillus* strains against seven antibiotics were evaluated using the standard disk diffusion method and are summarized in Table 2. Susceptibility was interpreted categorically as susceptible or resistant according to established criteria. The strains were identified as sensitive to cefazolin, penicillin, gentamicin, clarithromycin, and rifampicin, while exhibiting resistance to lincomycin and norfloxacin. These results indicate that the isolated strain shows a defined antibiotic sensitivity pattern, supporting its biosafety evaluation for potential application.

**TABLE 2:** Antibiotic susceptibility testing of *Lactobacillus* isolates.

Medication	Strain
	*Bacillus plantarum*
Cefazolin (30 μg/tablet)	S
Penicillin (10 μg /tablet)	S
Gentamicin (10 μg/tablet)	S
Clarithromycin (15 μg/tablet)	S
Lincomycin (2 μg/tablet)	R
Norfloxacin (10 μg/tablet)	R
Rifampicin (5 μg/tablet)	S

### 3.3 Animal safety evaluation

Supplementary Table S2, comparative observations of dietary intake and general behavior during the experimental period indicated that mice in Group B exhibited improved growth performance while maintaining normal food intake and mental status when compared with the control group (Group A).

**FIGURE 3:**
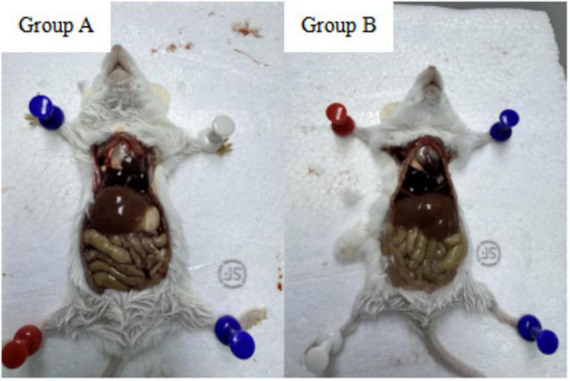
Macroscopic observation of visceral and intestinal tissues of mice in the **(A)** control group (administered physiological saline) and **(B)** experimental group (administered 1.0 × 10^8^ CFU/mL bacterial suspension for 2 weeks). No apparent abnormalities or lesions were observed in the tissues of the experimental group.

**FIGURE 4:**
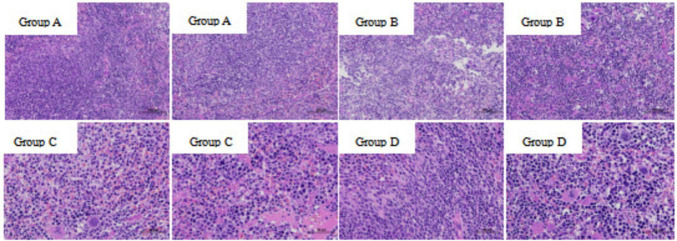
Histopathological examination of spleen tissues: **(A)** control group (200 × magnification); **(B)** experimental group (200 × magnification); **(C)** control group (400 × magnification); **(D)** experimental group (400 × magnification). No pathological changes, such as inflammation or necrosis, were observed in the spleens of mice treated with *L. plantarum*, indicating high *in vivo* safety.

Post-mortem examination revealed no visible abnormalities in visceral organs or intestinal tissues in either group. As shown in Figure 3, necropsy findings in the experimental group were comparable to those of the control group, indicating that oral administration of the isolated *Lactobacillus* strain at a concentration of 1.0 × 10^8^ CFU/mL did not induce pathological changes. Furthermore, histopathological analysis of spleen tissues using HE staining showed no observable abnormalities in the experimental group relative to the control group (Figure 4). These results collectively demonstrate that the isolated *Lactobacillus* strain exhibits a high level of safety in mice.

### 3.4 GPV titration and growth curve analysis in GEFs

On day 5 post-infection, control GEFs maintained normal morphology, whereas cells exposed to different concentrations of GPV exhibited clear and stable CPE. Viral titers were calculated using the Kärber method, and the results are presented in Table 3. GPV dilutions ranging from 10^−4^ to 10^−5^ produced approximately 50% CPE, yielding a viral titer of 10^4.5^ TCID_50_/100 μL for this GPV strain. Quantitative real-time PCR was used to determine Ct values at different time points following GPV infection. Using these values and the established standard curve (Figure 2C), the viral copy number in infected GEFs was calculated. The results showed that GPV replication peaked at 36 h post-infection, reaching approximately 10^5^.^22^ copies.

**TABLE 3:** Viral titers of GPV strains (TCID50/100 μL).

Virus solution dilution factor	Number of inoculation holes	Number of lesions	Incidence rate (%)
101	8	8	100%
102	8	8	100%
103	8	8	100%
104	8	5	62.5%
105	8	3	37.5%
106	8	0	0
107	8	0	0
108	8	0	0
109	8	0	0
1,010	8	0	0

### 3.5 Maximum safe dose of lactobacillus and its metabolites and their anti-GPV effects under different treatment strategies

**FIGURE 5:**
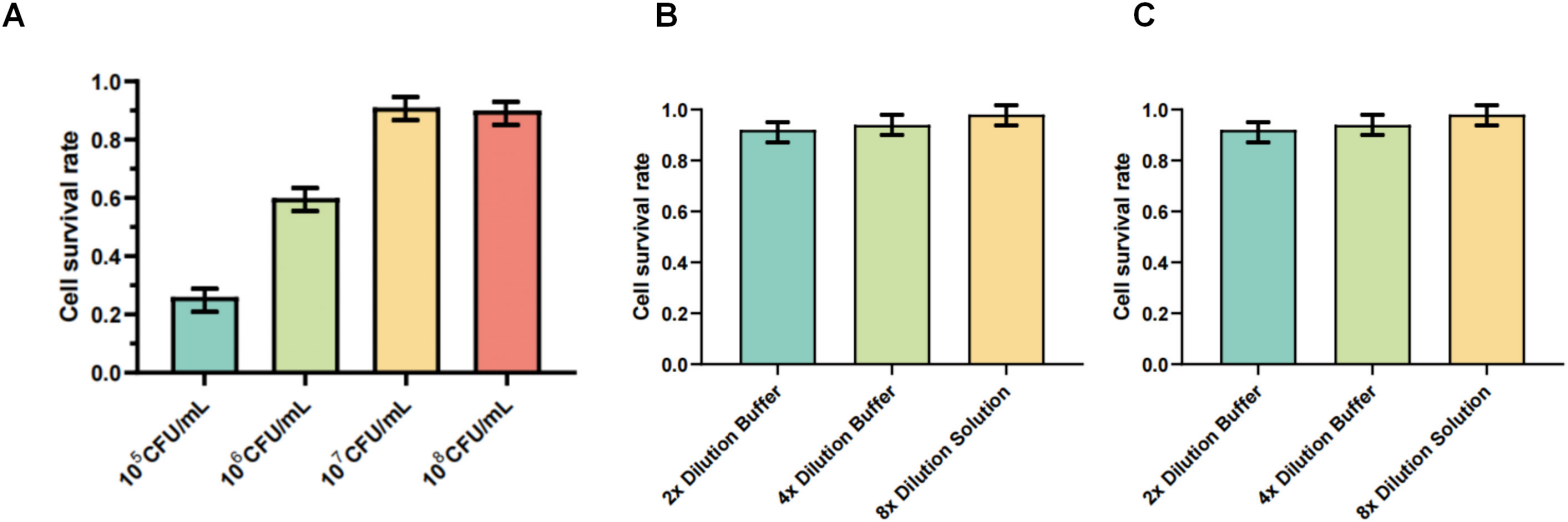
**(A)** GEFs were treated with serial dilutions of the bacterial suspension for 1.5 h. Cell viability was assessed 36 h later using the CCK-8 assay. The maximum safe concentration for GEFs was determined to be approximately 1.0 × 10^6^ CFU/mL. Data are presented as mean ± SD (*n* = 3). **(B)** GEFs were treated with serial dilutions of the cell-free metabolic supernatant. Cell viability was assessed using the CCK-8 assay after 36 h. The maximum safe concentration was a twofold dilution of the supernatant. Data are presented as mean ± SD (*n* = 3). **(C)** GEFs were treated with serial dilutions of heat-inactivated bacterial products. Cell viability was assessed using the CCK-8 assay after 36 h. The maximum safe concentration was a twofold dilution of the product. Data are presented as mean ± SD (*n* = 3).

As shown in Figures 5, the results of the CCK-8 assay indicated that the maximum safe concentration of the *Lactobacillus* suspension for GEFs was approximately 1.0 × 10^6^ CFU/mL. In contrast, both the heat-inactivated preparation and the metabolic supernatant exhibited maximum non-cytotoxic concentrations at a twofold dilution of the original 1.0 × 10^8^ CFU/mL suspension.

**FIGURE 6:**
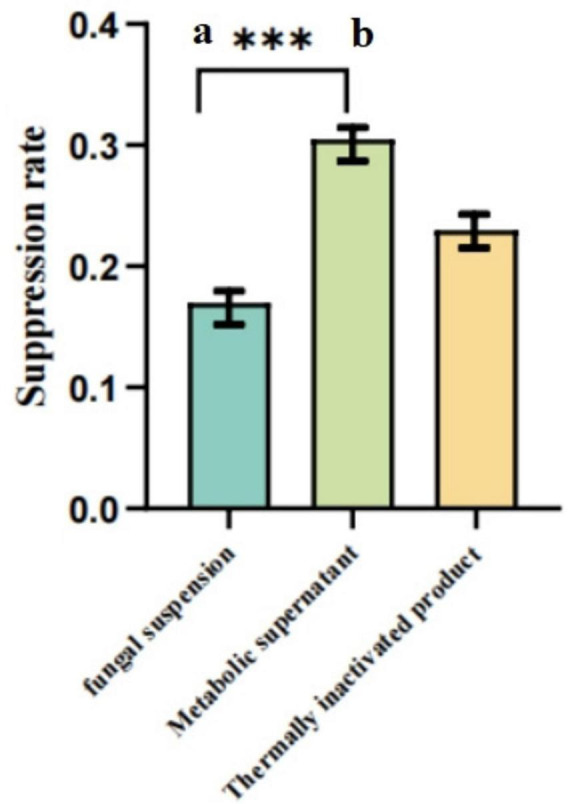
GEFs were pre-treated with the maximum safe concentration of each agent for 1.5 h before GPV infection. Viral inhibition rates were calculated based on cell viability determined by the CCK-8 assay compared to the virus control group. Data are presented as mean ± SD (*n* = 3). Different letters (a,b) indicate significant differences (*P* < 0.05) among groups. ***Significance is defined as a *p*-value less than 0.001.

**FIGURE 7:**
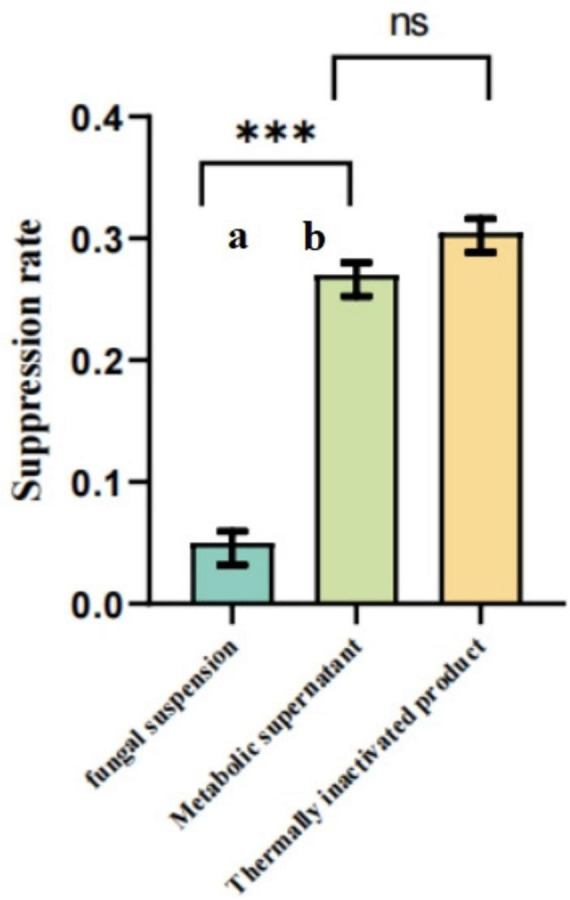
The agents were mixed with 100 TCID_50_ GPV and simultaneously added to GEFs for 1.5 h. Viral inhibition rates were assessed by the CCK-8 assay. Data are presented as mean ± SD (*n* = 3). Different letters (a,b) indicate significant differences (*P* <0.05) among groups. ***Significance is defined as a *p*-value less than 0.001.

**FIGURE 8:**
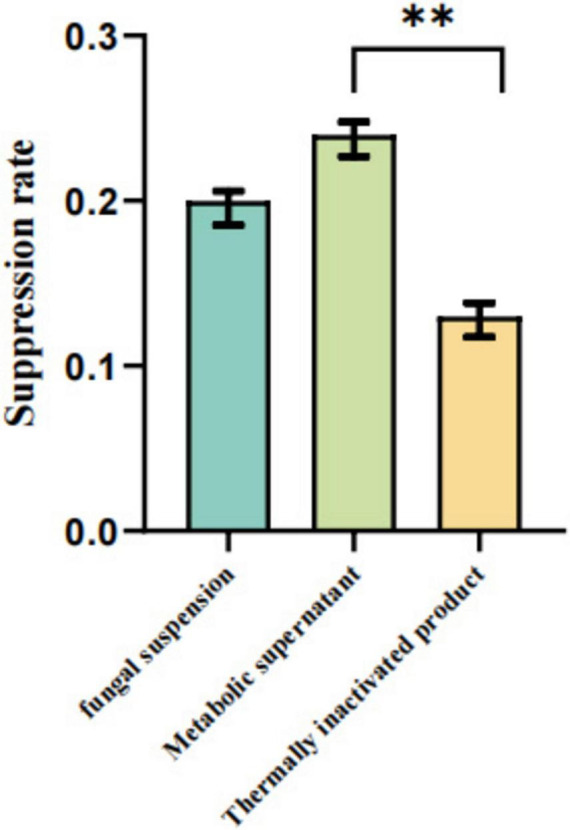
GEFs were first infected with GPV for 1.5 h, followed by treatment with the indicated agents. Viral inhibition rates were assessed by the CCK-8 assay. Data are presented as mean ± SD (*n* = 3). ** Indicates a highly significant difference (*P* < 0.01) between the metabolic supernatant and heat-inactivated product groups.

As illustrated in Figures 6–8, under both pretreatment and co-treatment conditions, the metabolic supernatant and heat-inactivated products of the isolated strain demonstrated higher GPV inhibition rates than the viable bacterial suspension. No significant differences were observed between the metabolic supernatant and heat-inactivated product groups under these two treatment conditions (*P* > 0.05). Under post-treatment conditions, however, the metabolic supernatant exhibited a significantly higher inhibitory effect against GPV compared with the heat-inactivated product (*P* < 0.01). Collectively, these results indicate that the antiviral effect against GPV is mainly attributable to secreted metabolites rather than viable bacterial cells.

### 3.6 Quantitative real-time PCR analysis of anti-GPV activity of bacterial suspensions and metabolic products

**FIGURE 9:**
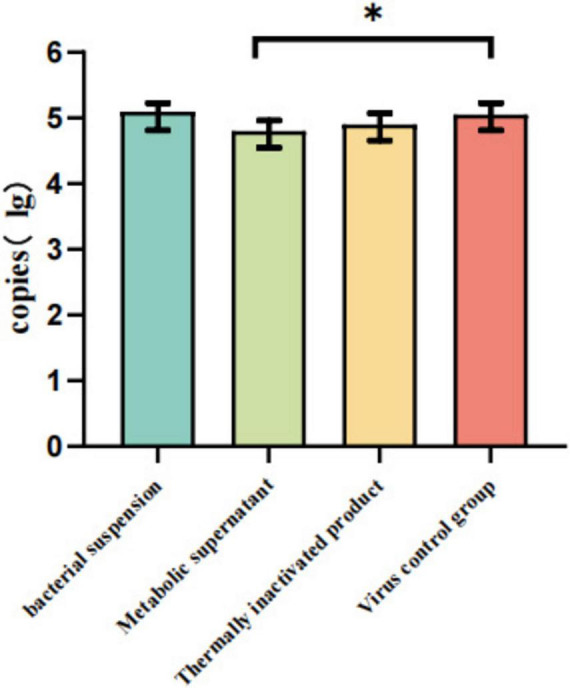
Each agent was mixed with 100 TCID_50_ GPV and incubated for 1.5 h. Viral RNA was extracted, and GPV copy numbers were quantified by qPCR. The metabolic supernatant group showed a significant reduction in GPV copies compared with the virus control group (*P < 0.05). Data are presented as mean ± SD (*n* = 3).

As shown in Figure 9, quantitative real-time PCR analysis revealed that treatment with the metabolic supernatant significantly reduced GPV copy numbers compared with the viral control group (*P* < 0.05). However, no statistically significant differences were detected among the bacterial suspension, metabolic supernatant, and heat-inactivated product treatment groups (*P* > 0.05).

### 3.7 Changes in active component content before and after fermentation of Chinese herbal decoction

#### 3.7.1 Chromatographic characteristics of reference standards and standard curve construction

**FIGURE 10:**
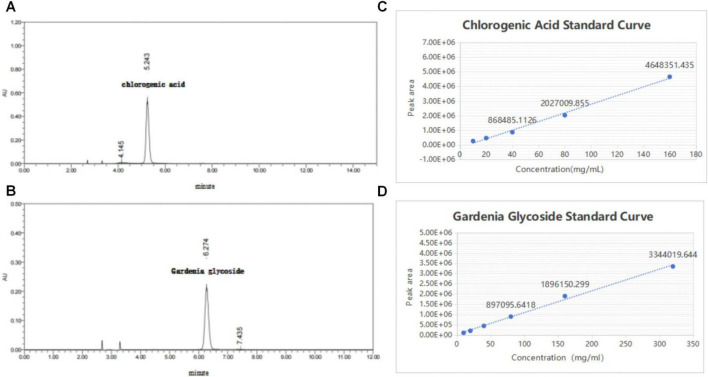
**(A)** Chromatographic conditions were as follows: column: (Column Type, e.g., C18); mobile phase: acetonitrile-0.4% phosphoric acid in water (13:87, v/v); flow rate: 0.1 mL/min; detection wavelength: 327 nm. The retention time for chlorogenic acid was 5.243 min. **(B)** Chromatographic conditions were as follows: column: (Column Type, e.g., C18); mobile phase: acetonitrile-water (15:85, v/v); flow rate: 1.0 mL/min; detection wavelength: 238 nm. The retention time for geniposide was 6.274 min. **(C)** A linear relationship was observed between peak area and chlorogenic acid concentration within the tested range. The regression equation was y = 2.92e+004x-1.59e+005, with *R*^2^ = 0.998667. **(D)** A linear relationship was observed between peak area and geniposide concentration within the tested range. The regression equation was y = 1.25e+004x-1.24e+005, with *R*^2^ = 0.992511.

As shown in Figures 10A,B the retention times of the chlorogenic acid and gardenoside reference standards were 5.243 and 6.274 min, respectively.

As illustrated in Figures 10C,D within the tested concentration ranges, the mass concentrations of chlorogenic acid and gardenoside exhibited good linear relationships with their corresponding peak areas.

#### 3.7.2 Determination of active components in Chinese herbal decoctions before and after fermentation

The retention times and peak shapes of the reference standards were compared with those of the test samples, and the peak areas of chlorogenic acid and gardenoside were determined. The concentrations of these active components in the TCM decoctions and their corresponding fermented products were calculated based on the standard curves. As shown in Table 4, compared with the unfermented herbal decoctions, the concentration of chlorogenic acid in the fermented honeysuckle decoction increased significantly, while the concentration of gardenoside in the fermented gardenia decoction also showed a significant increase.

**TABLE 4:** Changes in the content of active ingredients before and after fermentation of honeysuckle, gardenia and mixed decoction by isolated strains, respectively.

Detection components	Grouping	Peak area (microvolts per second)	Standard concentration conversion (μg/mL)
Chlorogenic acid	QZSL1 fermented honeysuckle decoction group	7061853.2048	247.2388
Chlorogenic acid	QZMR2 fermented honeysuckle decoction group	9665825.3641	336.3920
Chlorogenic acid	Honeysuckle decoction group (45 mL)	16627664.9150	255.4432
Gardenia glycoside	QZSL1 fermented gardenia decoction group	2178463.0987	160.0882
Gardenia glycoside	QZMR2 fermented gardenia decoction group	2821003.7192	204.0862
Gardenia glycoside	Gardenia decoction group	1025872.5616	81.1648
Chlorogenic acid	QZSL1 fermented mixed decoction group	4127142.8790	679.4666
Chlorogenic acid	QZMR2 fermented mixed decoction group	5076014.6965	831.1212
Chlorogenic acid	Combination decoction group	2357285.7239	396.5972

**FIGURE 11:**
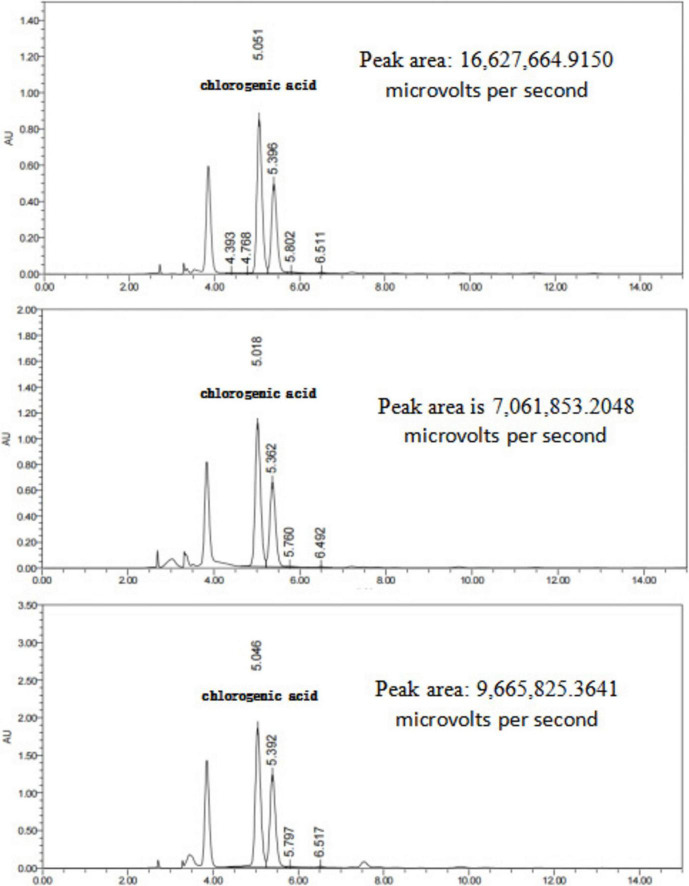
Representative HPLC of honeysuckle decoctions: (Bottom) unfermented honeysuckle decoction, (Middle) honeysuckle decoction fermented with strain QZSL1, and (Top) honeysuckle decoction fermented with strain QZMR2. The peak area of chlorogenic acid (Retention time: ∼5.243 min) was significantly increased after fermentation.

The HPLC chromatograms of the honeysuckle decoction, QZSL1-fermented honeysuckle decoction, and QZMR2-fermented honeysuckle decoction are presented in Figure 11.

**FIGURE 12:**
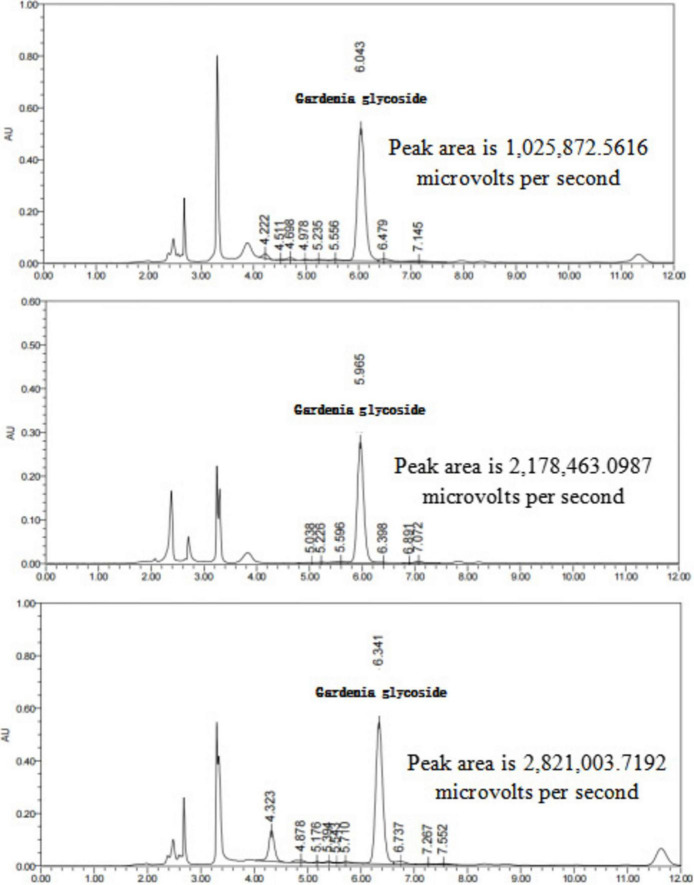
Representative HPLC chromatograms of gardenia decoctions: (Bottom) unfermented gardenia decoction, (Middle) gardenia decoction fermented with strain QZSL1, and (Top) gardenia decoction fermented with strain QZMR2. The peak area of geniposide (Retention time: ∼6.274 min) was significantly increased after fermentation.

The chromatograms of the gardenia decoction, QZSL1-fermented gardenia decoction, and QZMR2-fermented gardenia decoction are shown in Figure 12.

**FIGURE 13:**
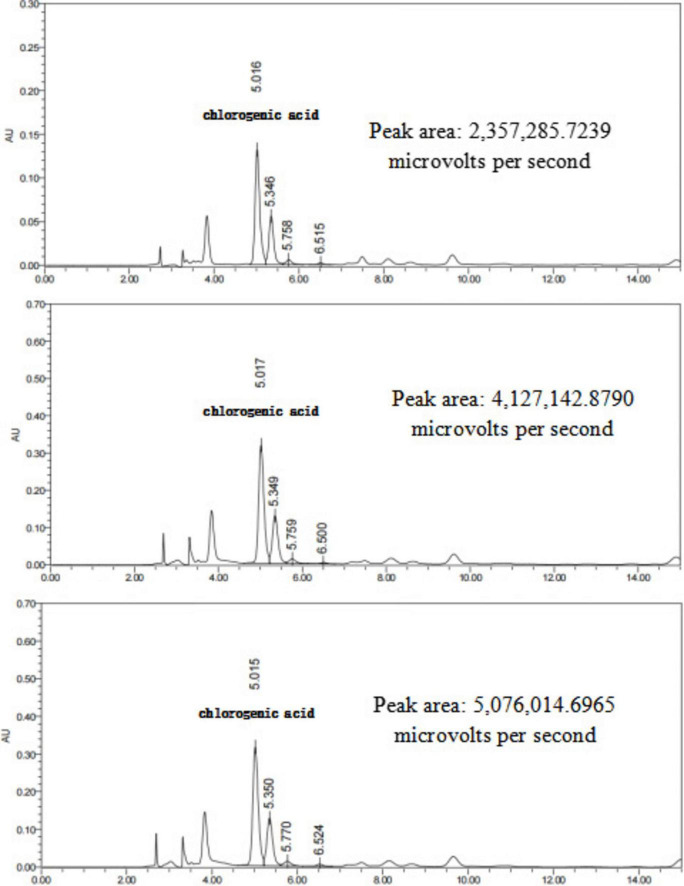
Representative HPLC chromatograms of mixed decoctions (honeysuckle + gardenia): (Bottom) unfermented mixed decoction, (Middle) mixed decoction fermented with strain QZSL1, and (Top) mixed decoction fermented with strain QZMR2. The peak area of chlorogenic acid was significantly increased after fermentation.

In addition, the chromatograms of the mixed decoction, QZSL1-fermented mixed decoction, and QZMR2-fermented mixed decoction are displayed in Figure 13. Collectively, these chromatographic analyses confirm that Lactobacillus-mediated fermentation improves the abundance of major active constituents in Chinese herbal decoctions.

### 3.8 Anti-GPV activity of lactobacillus-fermented Chinese herbal decoctions

#### 3.8.1 Determination of maximum safe concentrations of herbal decoctions and fermented products

**FIGURE 14:**
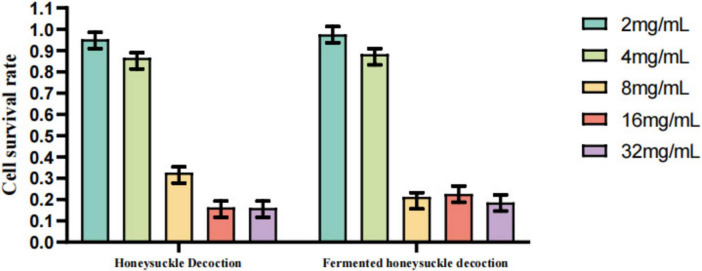
GEFs were treated with different concentrations (2–32 mg/mL) of honeysuckle decoctions for 1.5 h. Cell viability was assessed 48 h later using the CCK-8 assay. The maximum safe concentration for both was determined to be 4 mg/mL. Data are presented as mean ± SD (*n* = 3).

**FIGURE 15:**
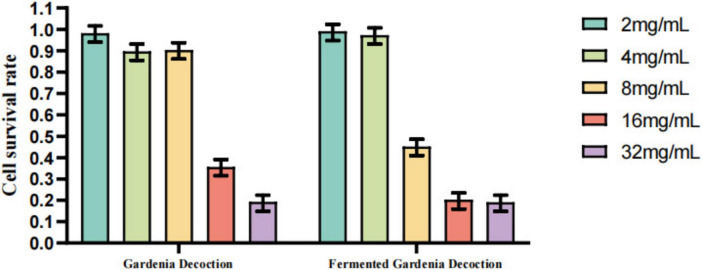
GEFs were treated with different concentrations (2–32 mg/mL) of gardenia decoctions. Cell viability was assessed by the CCK-8 assay. The maximum safe concentration of the unfermented gardenia decoction was 8 mg/mL, while that of the fermented decoction was 4 mg/mL. Data are presented as mean ± SD (*n* = 3).

As shown in Figures 14, 15 and Supplementary Figure S2, the results of the CCK-8 assay indicated that the maximum non-cytotoxic concentration of gardenia decoction and the mixed decoction in GEFs was 8 mg/mL. In contrast, the maximum safe concentration of honeysuckle decoction and fermented honeysuckle decoction was 4 mg/mL. The maximum safe concentration of fermented gardenia decoction was also 4 mg/mL, and that of the fermented mixed decoction was 4 mg/mL. Based on these results, a concentration of 4 mg/mL was selected for all herbal decoctions and fermented products in subsequent antiviral experiments.

#### 3.8.2 Anti-GPV effects of TCM decoctions and their fermented products

As shown in Supplementary Figures S3–S5, quantitative real-time PCR analysis demonstrated that, compared with the viral control group, treatment with fermented honeysuckle decoction, gardenia decoction, mixed decoction, and fermented gardenia decoction significantly reduced GPV copy numbers (*P* < 0.05). No significant difference was observed in the honeysuckle decoction group (*P* > 0.05). Notably, the fermented mixed decoction group exhibited a highly significant inhibitory effect against GPV (*P* < 0.001).

When the mixed decoction and fermented mixed decoction were applied using pretreatment, co-treatment, and post-treatment strategies, cell viability results presented in Supplementary Table S3 showed no significant difference in GPV inhibition rates between the two groups (*P* > 0.05). However, under both pretreatment and post-treatment conditions, the inhibition rates of GPV were significantly higher in the mixed decoction and fermented mixed decoction groups than those observed under the co-treatment condition. Overall, Lactobacillus fermentation markedly enhanced the anti-GPV efficacy of Chinese herbal decoctions, with the fermented mixed decoction exhibiting the strongest inhibitory effect.

### 3.9 Investigation of the mechanisms of action of TCMs against GPV based on network pharmacology and molecular docking

#### 3.9.1 Screening of active components and target molecules of *Lonicera japonica* and *Gardenia jasminoides*

A search of the TCMSP database using the keywords “honeysuckle” (*Lonicera japonica*) and “gardenia” (*Gardenia jasminoides*) yielded 23 and 15 candidate active components, respectively. All of these components met the predefined screening criteria for OB and DL. Among them, six components from honeysuckle and three from gardenia lacked corresponding target information and were therefore excluded. Ultimately, 17 active components from honeysuckle and 12 from gardenia were identified as effective components, corresponding to 455 and 366 target proteins, respectively. Target proteins were standardized using the UniProt database, and duplicate targets were removed, yielding the final target gene sets for subsequent analysis.

#### 3.9.2 Screening of potential GPV-related targets for *Lonicera japonica* and *Gardenia jasminoides*

A total of 170 GPV-related gene targets were collected from disease-related databases. Intersection analysis between the disease targets and the predicted targets of honeysuckle and gardenia was performed using EVenn software. Five overlapping target genes were identified, as shown in Supplementary Figure S6, and were considered potential therapeutic targets for further investigation.

#### 3.9.3 Construction of the drug-active component-target network

The intersecting target genes and their corresponding active components were imported into Cytoscape software to construct a drug–active component–target interaction network (Supplementary Figure S7). This network visually illustrates the relationships among TCMs, their active ingredients, and GPV-related targets. In the network, node size reflects the degree value, indicating the relative importance of each component or target.

As shown in Supplementary Table S4, analysis of the network revealed that quercetin, kaempferol, and β-sitosterol were the primary active components with the highest degree values.

#### 3.9.4 Construction of the GPV-related PPI network

The five intersecting target genes were imported into the STRING online database to construct a PPI network. Non-interacting targets were excluded, and the resulting interaction data were imported into Cytoscape software for visualization and further analysis, as shown in Supplementary Figure S8. Network topology analysis was performed using Cytoscape plugins to calculate node connectivity and identify core targets.

After filtering non-functional targets, core functional targets were screened based on node degree values. As summarized in Supplementary Table S5, the key target proteins involved in the anti-GPV effects of honeysuckle and gardenia included IL6, TNF, CASP3, IL-1β, PTGS2, BCL2, MMP9, ESR1, HIF1A, and TGFB1.

#### 3.9.5 GO function and KEGG pathway enrichment analysis

The intersecting target genes were imported into the DAVID database for GO functional and KEGG pathway enrichment analyses. For GO analysis, biological process (BP), cellular component (CC), and molecular function (MF) categories were selected. Enrichment results were filtered using thresholds of *P* < 0.01 and false discovery rate (FDR) < 0.01. GO terms and signaling pathways with higher count values were subsequently imported into the Microbioinformatics Platform visualization tool for graphical representation.

As shown in Supplementary Figure S9, GO functional enrichment analysis identified a total of 132 significantly enriched GO terms. Among these, seven terms were classified under cellular components, which were mainly enriched in transcription repressor complexes. Twenty-one terms were categorized under molecular functions, primarily associated with carboxylate ester surface activity, transcriptional activity, and protein kinase regulator activity. GO enrichment plots were generated by selecting the top-ranked terms based on *P*-values. Comprehensive analysis of these results indicated that the major targets of honeysuckle and gardenia in combating duck plague are predominantly localized in the extracellular space. Their pharmacological effects are mainly associated with the regulation of the G2/M phase transition of the mitotic cell cycle, immune responses, and carboxylate-mediated surface activity.

KEGG pathway enrichment analysis identified a total of 96 significantly enriched signaling pathways. As shown in Supplementary Figure S10, the top 20 pathways with *P* < 0.01 and the highest enrichment counts were selected to generate a pathway enrichment bubble chart. The results demonstrated that the Toll-like receptor signaling pathway, C-type lectin receptor signaling pathway, RIG-I-like receptor signaling pathway, and cytoplasmic DNA-sensing pathway are key signaling pathways through which honeysuckle and gardenia exert their therapeutic effects against duck plague.

#### 3.9.6 Molecular docking analysis results

The top four active compounds were individually docked with three core target proteins, and the docking results are summarized in Supplementary Table S6. The calculated binding free energies for all compound-target complexes were lower than –4.10 kcal/mol, indicating favorable binding interactions and strong affinity between the active compounds and their corresponding target proteins. Among these interactions, the β-sitosterol-TNF complex exhibited the lowest binding free energy, suggesting the highest structural stability.

As shown in Supplementary Figures S11, S12 the molecular docking conformations of quercetin–IL-6, kaempferol–IL-6, stigmasterol–IL-6, and stigmasterol–TNF were visualized using PyMOL software. The visualization results demonstrated that all active compounds were able to stably occupy the active binding pockets of the target proteins, forming favorable interactions, which further supports their potential roles in mediating the anti-GPV effects of honeysuckle and gardenia.

Taken together, the network pharmacology and molecular docking analyses provide an integrated mechanistic framework for the anti-GPV effects of Lonicera japonica and Gardenia jasminoides. Screening of active components and target prediction revealed that these two herbs exert their antiviral effects through a multi-component, multi-target mode of action. Key bioactive compounds, including quercetin, kaempferol, and β-sitosterol, were identified as central nodes in the drug–component–target network, indicating their dominant roles in mediating therapeutic activity. Intersection analysis further highlighted a limited but functionally critical set of GPV-related targets, which were enriched in immune regulation, inflammatory responses, apoptosis, and antiviral defense pathways. GO and KEGG enrichment analyses consistently demonstrated that the therapeutic effects of honeysuckle and gardenia are closely associated with innate immune signaling pathways, including Toll-like receptor, C-type lectin receptor, RIG-I-like receptor, and cytoplasmic DNA-sensing pathways, suggesting a coordinated modulation of host antiviral immunity. Moreover, molecular docking results confirmed favorable binding interactions between the major active compounds and core target proteins such as IL-6 and TNF, providing structural support for the predicted network interactions. Collectively, these findings suggest that honeysuckle and gardenia combat GPV infection by regulating host immune and inflammatory signaling through multiple bioactive constituents acting on key antiviral targets.

## 4 Discussion

In this study, a *Lactobacillus plantarum* strain was systematically isolated and identified from silage feed and subsequently applied to the fermentation of TCMs, including honeysuckle and gardenia. The antiviral activity of the resulting fermentation products against GPV was comprehensively investigated, with the aim of elucidating the underlying mechanisms involved. The results confirmed that the isolated strain possesses excellent probiotic characteristics, including strong tolerance to acidic and bile salt environments, inhibitory activity against common pathogenic bacteria, and good biosafety (Hopkins, 2008). Furthermore, the findings demonstrated that fermentation with *L. plantarum* significantly enhanced the contents of key active components and improved the antiviral efficacy of the Chinese herbal medicines (Forli et al., 2016). In addition, the application of network pharmacology and molecular docking approaches enabled preliminary identification of potential target proteins and signaling pathways associated with the observed antiviral effects. These results provide a theoretical basis and practical reference for the development and application of fermented Chinese herbal medicines in the prevention and control of viral infections (Yao et al., 2020).

Microbial fermentation of TCM represents a novel integration of traditional Chinese medical theory with modern biotechnology and has attracted increasing attention in recent years (Yang et al., 2023 ). In the present study, honeysuckle and gardenia were selected as fermentation substrates due to their well-documented antiviral, anti-inflammatory, and immunomodulatory properties. Their major bioactive constituents, chlorogenic acid and gardenoside, have been confirmed by numerous studies to possess broad biological activities (Shang et al., 2011; Xiao et al., 2017). However, the pharmacological potential of active compounds in raw herbal materials is often limited by factors such as high molecular weight, poor solubility, and low bioavailability. Lactic acid bacteria fermentation facilitates the biotransformation of herbal matrices through diverse enzymatic systems, including glycosidases and esterases. This process can disrupt plant cell wall structures, thereby promoting the release of encapsulated active components. Moreover, microbial metabolism may generate novel bioactive compounds or reduce the toxicity of certain original constituents (Liu et al., 2020). In the present study, HPLC analysis demonstrated significant increases in chlorogenic acid and gardenoside contents following fermentation, indicating that microbial fermentation is an effective strategy for enhancing the efficacy of TCM (Zhang et al., 2023). This enhancement likely results not only from physical release of active substances but also from biocatalytic transformations, such as the hydrolysis of glycosides mediated by *Lactobacillus* β-glucosidase activity, which may improve bioavailability and ultimately enhance therapeutic effects (Vavers et al., 2023).

With respect to antiviral mechanisms, this study represents a pioneering effort to integrate fermented TCMs into the prevention and control of GPV, thereby addressing a critical gap in the application of fermented herbal medicines for avian viral diseases. *In vitro* cell-based experiments demonstrated that metabolic extracts of *L. plantarum* significantly inhibited GPV replication, with particularly pronounced effects observed in pretreatment and co-treatment models. These findings suggest potential mechanisms involving direct inhibition of viral adsorption or entry, as well as modulation of host cell antiviral states (Al Kassaa et al., 2019). Further qPCR analysis of viral copy numbers confirmed the strong antiviral efficacy of the fermented mixed decoction. This effect may be attributed to increased concentrations of bioactive compounds and fermentation-derived metabolites, including organic acids and bacteriocins. Organic acids, such as lactic acid and acetic acid, have been reported to destabilize viral structures by lowering the surrounding pH, thereby impairing viral stability and infectivity (Tullman et al., 2020). In addition, bacteriocins may exert antiviral effects by directly disrupting viral particles or interfering with viral replication processes (Riester et al., 2021). Moreover, fermentation-derived metabolites, including extracellular polysaccharides and peptides, have been shown to possess immunomodulatory properties, which may indirectly enhance host antiviral responses (Zhu et al., 2022).

Network pharmacology analysis revealed that the honeysuckle-gardenia fermentation system exhibits typical multi-component, multi-target, and multi-pathway characteristics in its anti-GPV activity (Li et al., 2023). In the present study, several compounds were identified as core active constituents, including quercetin, kaempferol, β-sitosterol, and stigmasterol. These bioactive components were found to act on key inflammation- and apoptosis-related targets, such as IL-6, TNF, CASP3, and IL-1β. Furthermore, pathway enrichment analysis indicated significant involvement of immune-related signaling pathways, including the Toll-like receptor signaling pathway and the C-type lectin receptor signaling pathway (Zhang et al., 2019). Collectively, these findings suggest that fermented Chinese herbal medicines may influence viral replication and transmission through both direct and indirect mechanisms. Direct effects may be mediated by modulation of host immune and inflammatory responses, while indirect effects may occur through the coordinated regulation of these pathways, ultimately enhancing antiviral defense (Akira and Takeda, 2004). Molecular docking analysis further validated the strong binding affinity between the identified active components and their corresponding target proteins at the structural level. Notably, β-sitosterol exhibited a particularly low binding free energy with TNF (-7.5 kcal/mol), indicating a highly stable interaction and strong potential for biological activity (Iwasaki and Pillai, 2014). This observation provides a molecular basis for understanding the antiviral mechanisms of fermented Chinese herbal medicines and offers a theoretical foundation for future targeted drug design.

It should be noted that although this study demonstrated the favorable safety profile and antiviral efficacy of fermented Chinese herbal medicines *in vitro* and in murine models, their effectiveness under actual farming conditions requires further validation (Forli et al., 2016). In addition, safety evaluations were conducted exclusively in mice, without the inclusion of natural GPV hosts such as geese or Muscovy ducks. Therefore, caution is warranted when extrapolating these findings to practical applications in poultry production systems (Meng et al., 2022). Furthermore, several technical challenges must be addressed to facilitate future translational application. These include standardization of fermentation processes, stabilization of active components, and the establishment of robust quality control systems for large-scale production (Hou et al., 2023). For example, microbial metabolic activity during fermentation can be influenced by multiple factors, such as medium composition, pH, and temperature. Consequently, systematic optimization of fermentation conditions—potentially through approaches such as response surface methodology—is necessary. In parallel, further studies are required to evaluate the storage stability of fermentation products, their *in vivo* pharmacokinetic properties, and long-term safety profiles.

In summary, this study successfully isolated a *L. plantarum* strain with excellent probiotic characteristics and systematically evaluated its antiviral activity and underlying mechanisms through the fermentation of traditional Chinese herbal medicines. The findings provide important experimental evidence supporting the development of novel biological agents as alternatives to antibiotics. As a green and safe strategy, fermented Chinese herbal medicines have demonstrated the ability to enhance therapeutic efficacy, improve intestinal health, and inhibit viral transmission. Future research should focus on comprehensive *in vivo* efficacy evaluation, dose optimization, deeper mechanistic exploration, and the development of scalable industrial production processes. Addressing these priorities will facilitate the practical application of fermented Chinese herbal medicines and help meet the growing demands of green animal husbandry and animal disease prevention. Through interdisciplinary integration and technological innovation, fermented Chinese herbal medicines show strong potential as a new generation of functional feed additives for controlling viral diseases in livestock and poultry, offering both theoretical significance and practical value.

## 5 Conclusion

1. A *Lactobacillus plantarum* strain with excellent probiotic properties was successfully isolated and identified in this study;
2. The bacterial suspension, cell-free supernatant, and heat-inactivated products of the isolated *Lactobacillus* strain all exhibited anti-GPV activity, with the metabolic supernatant showing the strongest inhibitory effect;
3. The fermented mixed decoction demonstrated a highly significant anti-GPV effect. Although no significant difference (*P* > 0.05) was observed between the decoction group and the fermented decoction group in terms of GPV inhibition rates, both treatments achieved inhibition rates exceeding 50%.
4. The primary active components of honeysuckle and gardenia against GPV include quercetin, kaempferol, β-sitosterol, and stigmasterol. These compounds exert antiviral effects through multiple signaling pathways, including Toll-like receptor signaling, C-type lectin receptor signaling, RIG-I-like receptor signaling, and cytoplasmic DNA-sensing pathways. Key target molecules involved in these processes include IL-6, TNF, CASP3, and IL-1β, among others.

## Data availability statement

The raw data supporting the conclusions of this article will be made available by the authors, without undue reservation.
