# Correction: Anti-GPV activity of *Lactobacillus*-fermented traditional Chinese medicines

**DOI:** 10.3389/fmicb.2026.1890688

**Published:** 2026-07-06

**Authors:** Hongrui Chen, Songrui Liu, Qiuxuan Wang, Hanjia Zhang, Meng Qingfeng, Hao Dong

**Affiliations:** 1 College of Life Sciences, Jilin Agricultural University, Changchun, Jilin, China; 2 Ministry of Education Engineering Research Center for Bioreactors and Drug Development, College of Life Sciences, Jilin Agricultural University, Changchun, Jilin, China

**Keywords:** antiviral activity, bioconversion, fermented Chinese medicinal materials, GPV, metabolites, microbial fermentation, network, pharmacology

1. The reference for [Acosta-González et al., 2015] was erroneously written as [Acosta-González, A., Martirani-von Abercron, S. M., Rosselló-Móra, R., Wittich, R. M., Marqués, S. (2015). The effect of oil spills on the bacterial diversity and catabolic function in coastal sediments: a case study on the Prestige oil spill. Environ. Sci. Pollut. Res. 22:15200–15214. doi: 10.1007/s11356-015-4458-y]. It should be [Li, J., Feng, S., Liu, X., Jia, X., Qiao, F., Guo, J., et al. (2022). Effects of traditional Chinese medicine and its active ingredients on drug-resistant bacteria. *Front. Pharmacol*. 13:837907. doi: 10.3389/fphar.2022.837907].

2. The reference for [Araújo et al., 2021] was erroneously written as [Araújo, F.F., Farias, D.P., Neri-Numa, I.A., Pastore, G.M. (2021). Underutilized plants of the Cactaceae family: Nutritional aspects and technological applications. Food Chem. 362:130196. doi: 10.1016/j.foodchem.2021.130196]. It should be [Zhang, X., Miao, Q., Pan, C., Yin, J., Wang, L., Qu, L., et al. (2023). Research advances in probiotic fermentation of Chinese herbal medicines. *Imeta* 2:e93. doi: 10.1002/imt2.93].

3. The reference for [Bilal et al., 2024] was erroneously written as [Bilal, S., Tariq, A., Khan, S.I., Liaqat, M., Andreescu, S., Zhang, H., Hayat, A. (2024). A review of nanophotonic structures in optofluidic biosensors for food safety and analysis. Trends Food Sci. Technol. 147:104428. doi: 10.1016/j.tifs.2024.104428]. It should be [Li, L., Yang, L., Yang, L., He, C., He, Y., Chen, L., et al. (2023). Network pharmacology: a bright guiding light on the way to explore the personalized precise medication of traditional Chinese medicine. *Chin. Med*. 18:146. doi: 10.1186/s13020-023-00853-2].

4. The reference for [Dhakal et al., 2020] was erroneously written as [Dhakal, P., Chettri, B., Lepcha, S., Acharya, B.K. (2020). Rich yet undocumented ethnozoological practices of socio-culturally diverse indigenous communities of Sikkim Himalaya, India. J. Ethnopharmacol. 249:112386. doi: 10.1016/j.jep.2019.112386] It should be [Chen, G., Li, Z., Liu, S., Tang, T., Chen, Q., Yan, Z., et al. (2023). Fermented Chinese herbal medicine promoted growth performance, intestinal health, and regulated bacterial microbiota of weaned piglets. *Animals* 13:476. doi: 10.3390/ani13030476].

5. The reference for [Fang et al., 2025] was erroneously written as [Fang, Y., Zhu, Z., Xie, C., Xia, D., Zhao, H., Wang, Z., Lu, Q., Zhang, C., Xiong, W., Yang, X. (2025). Design and synthesis of novel saponin-triazole derivatives in the regulation of adipogenesis. Chin. J. Nat. Med. 23:920-931. doi: 10.1016/S1875-5364(25)60830-2]. It should be [Ruan, M., Zhang, L. Y., Yu, B., Chen, Y. S., and Zhuang, Y. (2010). Transformation of astragaloside IV in bidirectional solid fermenting of *Astragalus membranaceus*. *Zhong Yao Cai* 33, 339–343].

6. The reference for [Miles et al., 2023] was erroneously written as [Miles, S.L., Torraca, V., Dyson, Z.A., López-Jiménez, A.T., Foster-Nyarko, E., Lobato-Márquez, D., Jenkins, C., Holt, K.E., Mostowy, S. (2023). Acquisition of a large virulence plasmid (pINV) promoted temperature-dependent virulence and global dispersal of O96:H19 enteroinvasive Escherichia coli. mBio 14:e00882-23. doi: 10.1128/mbio.00882-23]. It should be [Yang, H. Y., Han, L., Lin, Y. Q., Li, T., Wei, Y., Zhao, L. H., et al. (2023). Probiotic fermentation of herbal medicine: progress, challenges, and opportunities. *Am. J. Chin. Med*. 51, 1105–1126. doi: 10.1142/S0192415X23500519].

7. The reference for [Peng et al., 2021] was erroneously written as [Peng, R., Jones, D.C., Liu, F., Zhang, B. (2021). From sequencing to genome editing for cotton improvement. Trends Biotechnol. 39:221-224. doi: 10.1016/j.tibtech.2020.09.001]. It should be [Shang, X., Pan, H., Li, M., Miao, X., and Ding, H. (2011). *Lonicera japonica* Thunb.: ethnopharmacology, phytochemistry and pharmacology of an important traditional Chinese medicine. *J. Ethnopharmacol*. 138, 1–21. doi: 10.1016/j.jep.2011.08.016] and [Xiao, W., Li, S., Wang, S., and Ho, C. T. (2017). Chemistry and bioactivity of *Gardenia jasminoides*. *J. Food. Drug. Anal*. 25, 43–61. doi: 10.1016/j.jfda.2016.11.005].

8. The reference for [Tomasello et al., 2024] was erroneously written as [Tomasello, L., Holub, S.M., Nigita, G., Distefano, R., Croce, C.M. (2024). Poly(A)-specific RNase (PARN) generates and regulates miR-125a-5p 3'-isoforms, displaying an altered expression in breast cancer. Signal Transduct. Target. Ther. 9:90. doi: 10.1038/s41392-024-01795-3]. It should be [Zhu, H., Guo, L., Yu, D., and Du, X. (2022). New insights into immunomodulatory properties of lactic acid bacteria fermented herbal medicines. *Front. Microbiol*. 13:1073922. doi: 10.3389/fmicb.2022.1073922].

9. The reference for [Lin et al., 2019] was erroneously written as [Lin, L.T., Hsu, W.C., Lin, C.C. (2019). Antiviral natural products and herbal medicines. J. Tradit. Complement. Med. 9:1-7. doi: 10.1016/j.jtcme.2018.02.001]. It should be [Chen, Z., and Ye, S. Y. (2022). Research progress on antiviral constituents in traditional Chinese medicines and their mechanisms of action. *Pharm. Biol*. 60, 1063–1076. doi: 10.1080/13880209.2022.2074053].

The original version of this article has been updated.

