# Supplementary material for: Anti-GPV activity of Lactobacillus-fermented traditional Chinese medicines
Source: Front Microbiol. 2026 Feb 10;17:1738123. doi: 10.3389/fmicb.2026.1738123 (PMC12929476; doi:10.3389/fmicb.2026.1738123)
Supplement: Supplementary file 1 [file Presentation_1.pdf]

Supplementary figure S1. Genomic DNA of the isolated strain was extracted and used as a template for PCR amplification with universal 16S rRNA primers. The amplified product was approximately 1500 bp, as shown by electrophoresis on a 1% agarose gel. Lane M: DNA molecular weight marker.

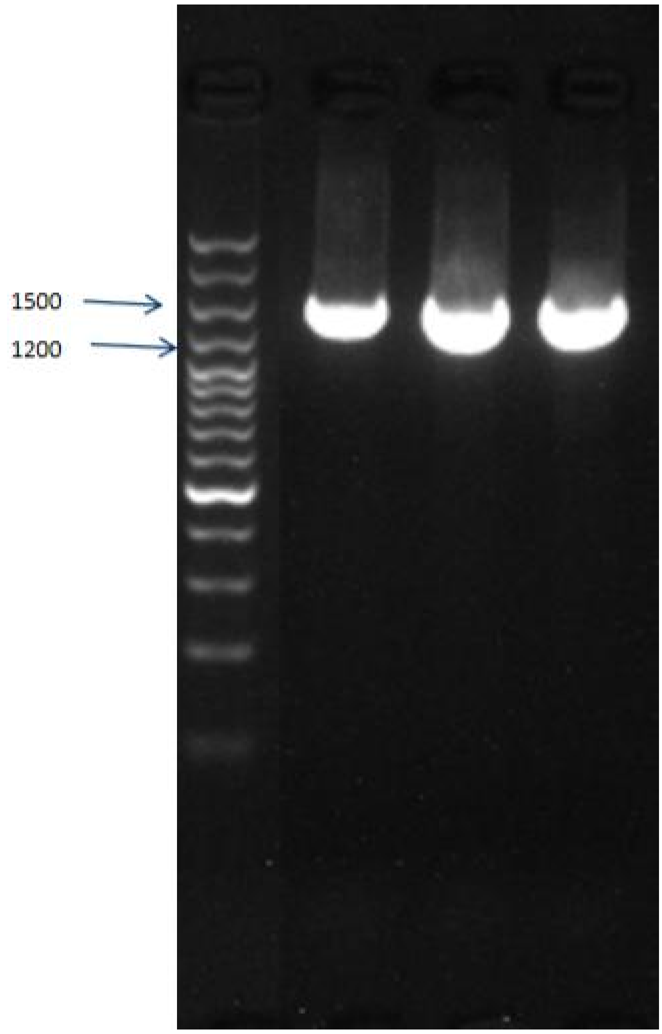

Supplementary figure S2.GEFs were treated with different concentrations (2-32 mg/mL) of the decoctions. Cell viability was assessed by the CCK-8 assay. The maximum safe concentration of the unfermented mixed decoction was 8 mg/mL, while that of the fermented mixed decoction was 4 mg/mL. Data are presented as mean ± SD (n=3).

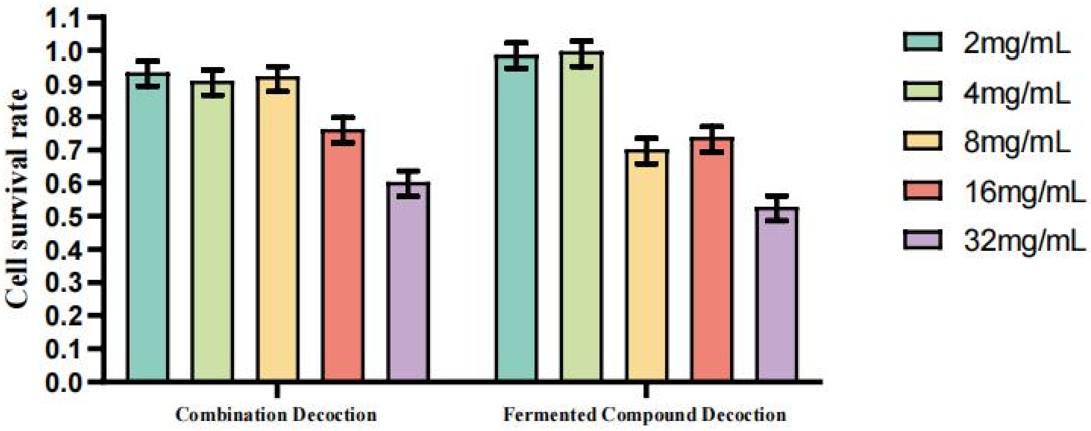

Supplementary figure S3. GEFs were treated with the maximum safe concentration (4 mg/mL) of each decoction mixed with 100 $TCID_{50}$ GPV for 1.5 hours. Viral copy numbers were quantified by qPCR after 36 hours. The fermented honeysuckle decoction group showed significantly reduced GPV copies compared to the virus control group (*$P < 0.05$). Data are presented as mean ± SD (n=3).

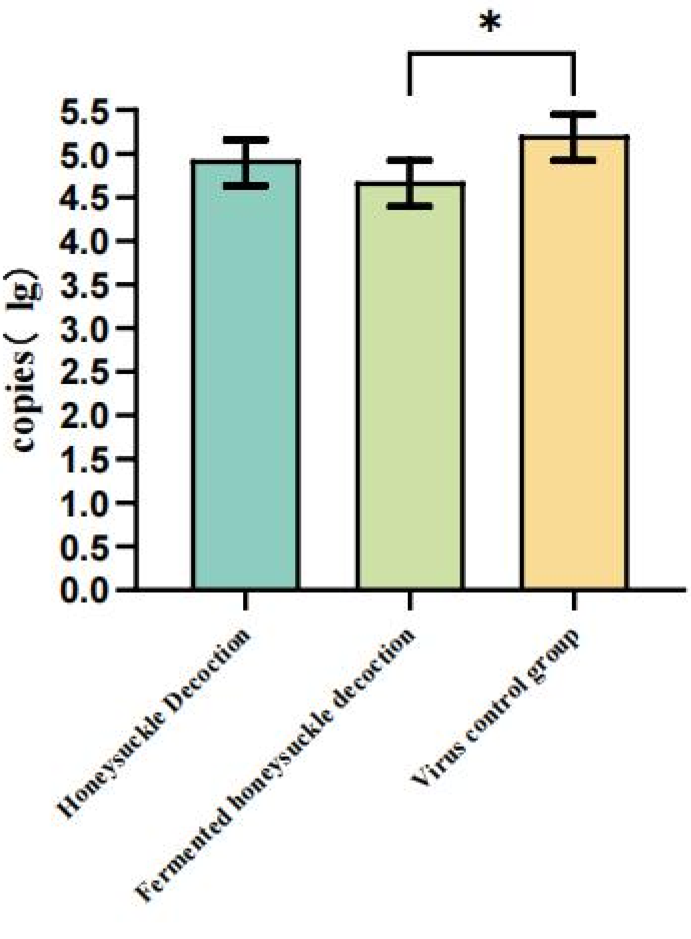

Supplementary figure S4. The experimental procedure was identical to that described in Supplementary figure S3. Both the unfermented and fermented gardenia decoction groups showed significantly reduced GPV copy numbers (*$P < 0.05$). Data are presented as mean ± SD (n=3).

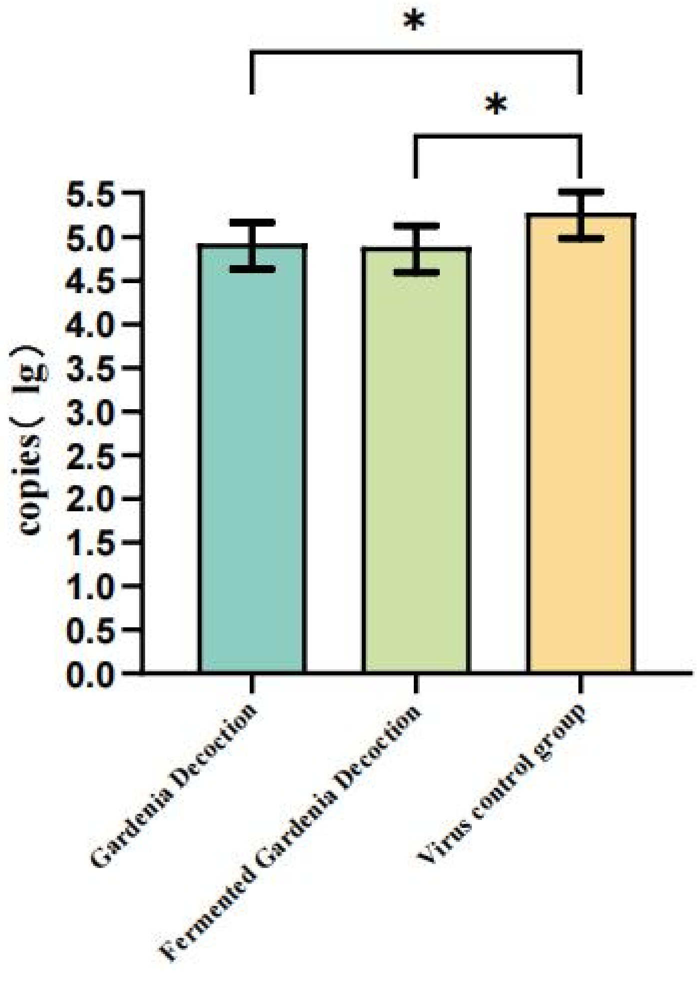

Supplementary figure S5. The experimental procedure was identical to that described in Supplementary figure S3. The fermented mixed decoction group exhibited an extremely significant anti-GPV effect (***$P < 0.001$). Data are presented as mean ± SD (n=3).

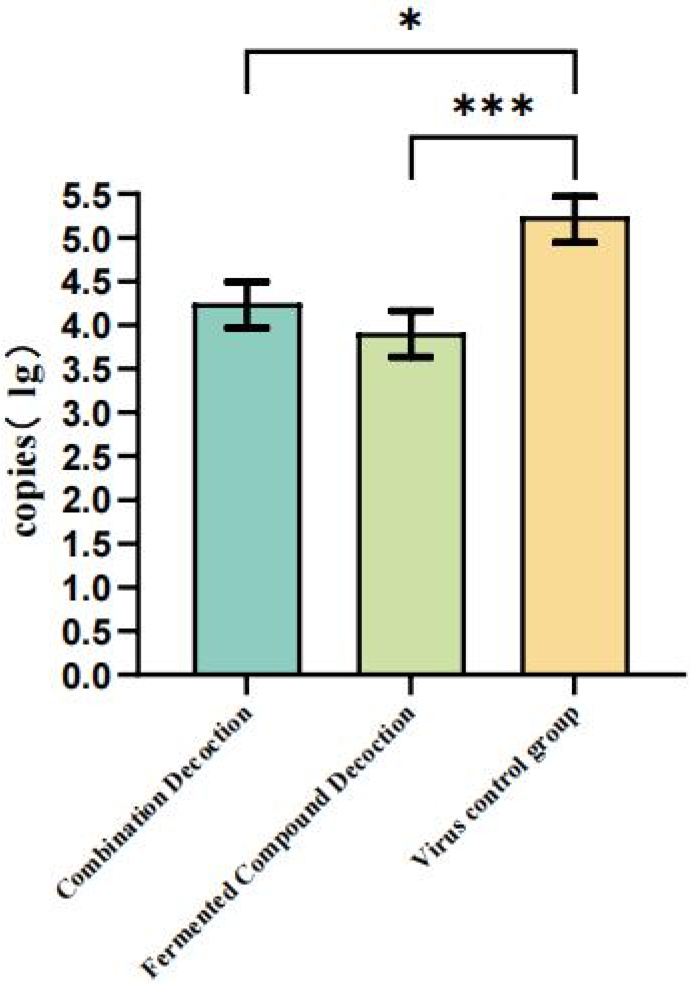

Supplementary figure S6. Venn diagram showing the overlap between predicted targets of honeysuckle (455 targets), gardenia (366 targets), and GPV-related targets (170 targets). A total of five common targets were identified.

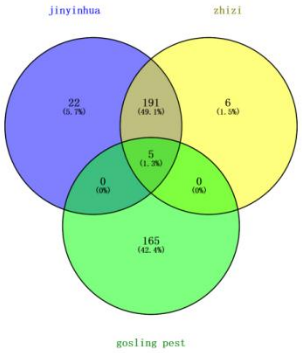

Supplementary figure S7. rug–active ingredient–target interaction network constructed using Cytoscape software. Green nodes represent active ingredients of honeysuckle; blue nodes represent active ingredients of gardenia; yellow nodes represent common ingredients; diamond-shaped nodes represent potential targets. Node size is proportional to the degree of connectivity.

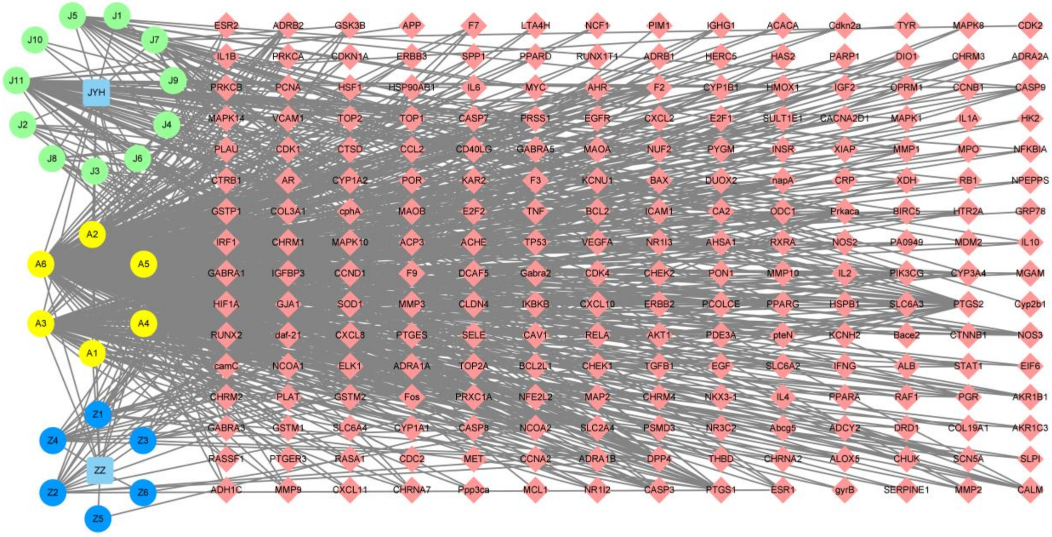

Supplementary figure S8. Protein–protein interaction (PPI) network of the 5 common targets constructed using the STRING database and visualized with Cytoscape. The network reveals the interactions among key targets, including IL6, TNF, and CASP3.

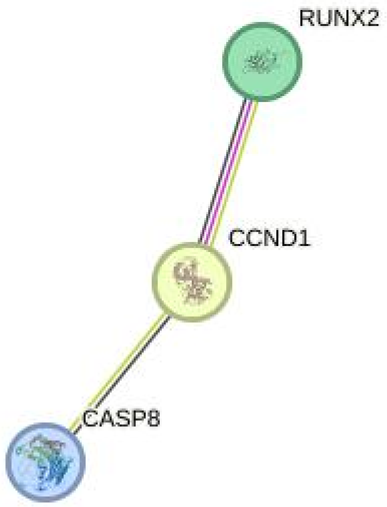

Supplementary figure S9. The bar chart shows the top enriched terms in biological process (BP), cellular component (CC), and molecular function (MF) categories. The key targets are primarily involved in regulating the mitotic cell cycle G2/M transition, immune response, and carboxylic ester hydrolase activity.

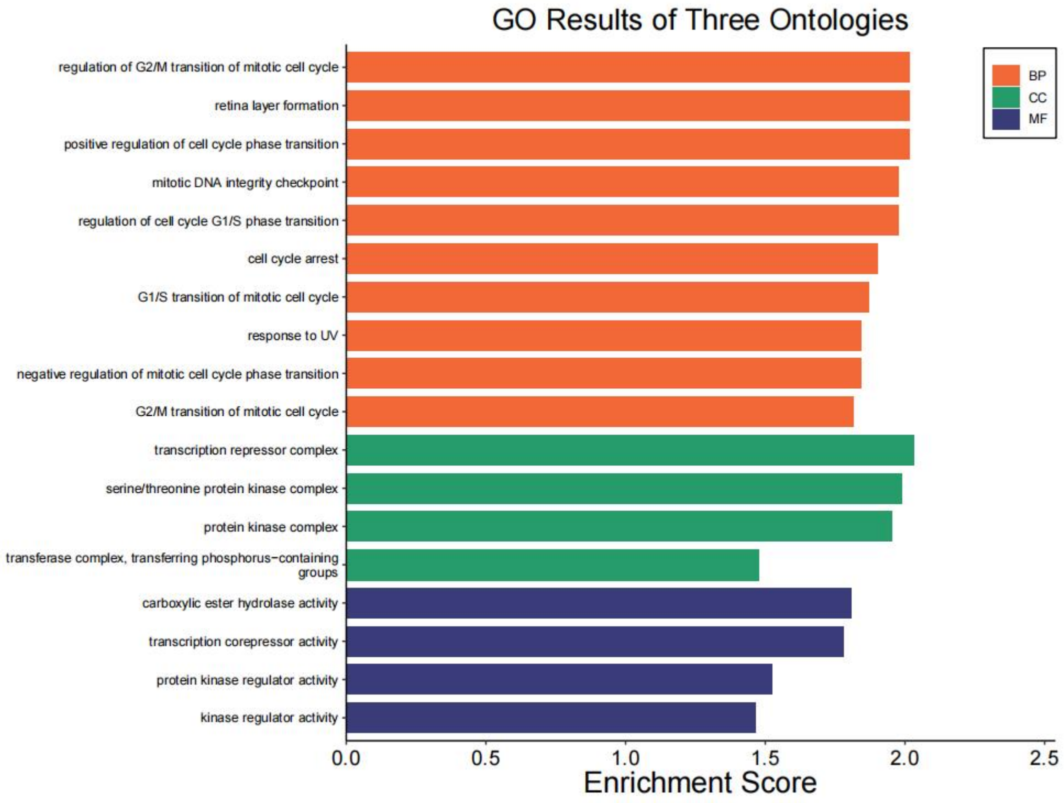

Supplementary figure S10. The bubble chart shows the top 20 significantly enriched KEGG pathways. Bubble size represents the number of genes, and color represents the -log10(*P*-value). Key pathways include the Toll-like receptor signaling pathway, the C-type lectin receptor signaling pathway, and the RIG-I-like receptor signaling pathway.

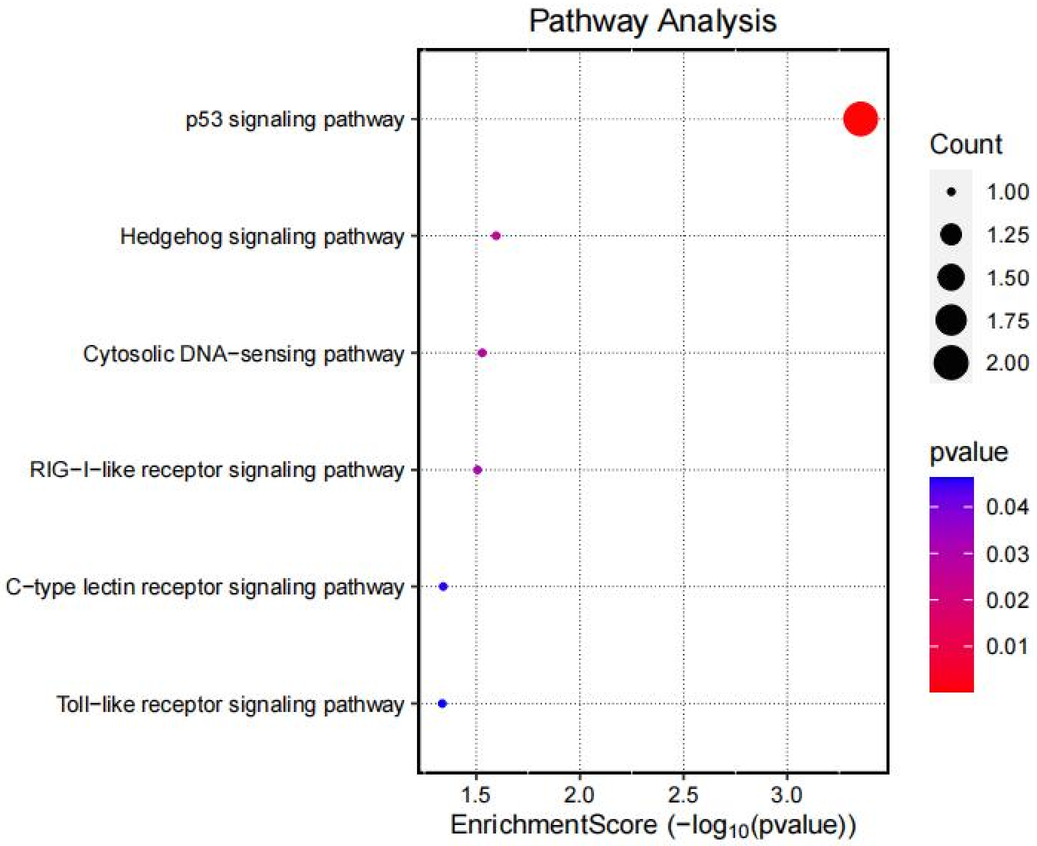

Supplementary figure S11. 2D visualization of the molecular docking interactions: (A) Quercetin with IL-6; (B) Kaempferol with IL-6; (C) Stigmasterol with IL-6; (D) Stigmasterol with TNF. The active compounds form stable interactions, including hydrogen bonds and hydrophobic interactions, within the binding pockets of the target proteins.

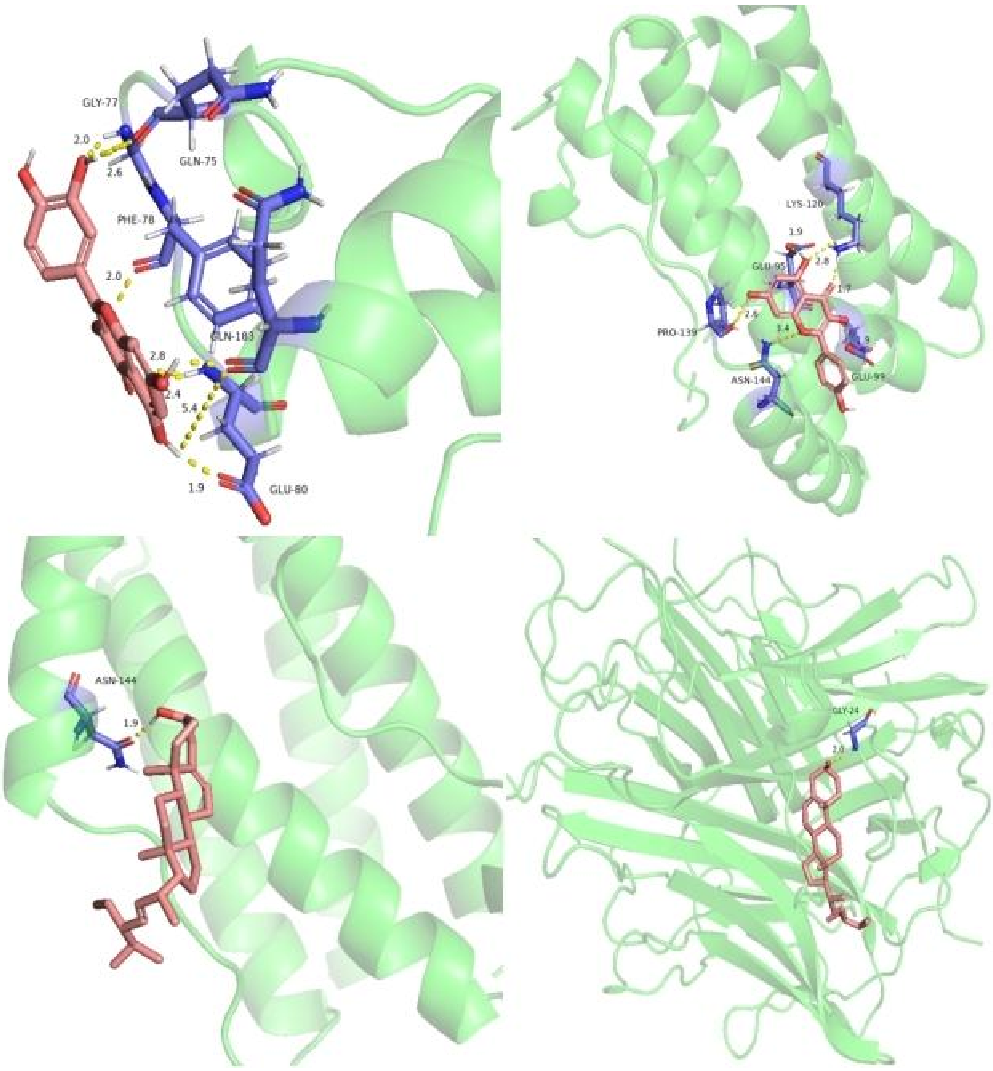

Supplementary figure S12. 3D surface representations of molecular docking poses: (A) Quercetin with IL-6; (B) Kaempferol with IL-6; (C) Stigmasterol with IL-6; (D) Stigmasterol with TNF. The active compounds are shown stably occupying the binding pockets of the target proteins.

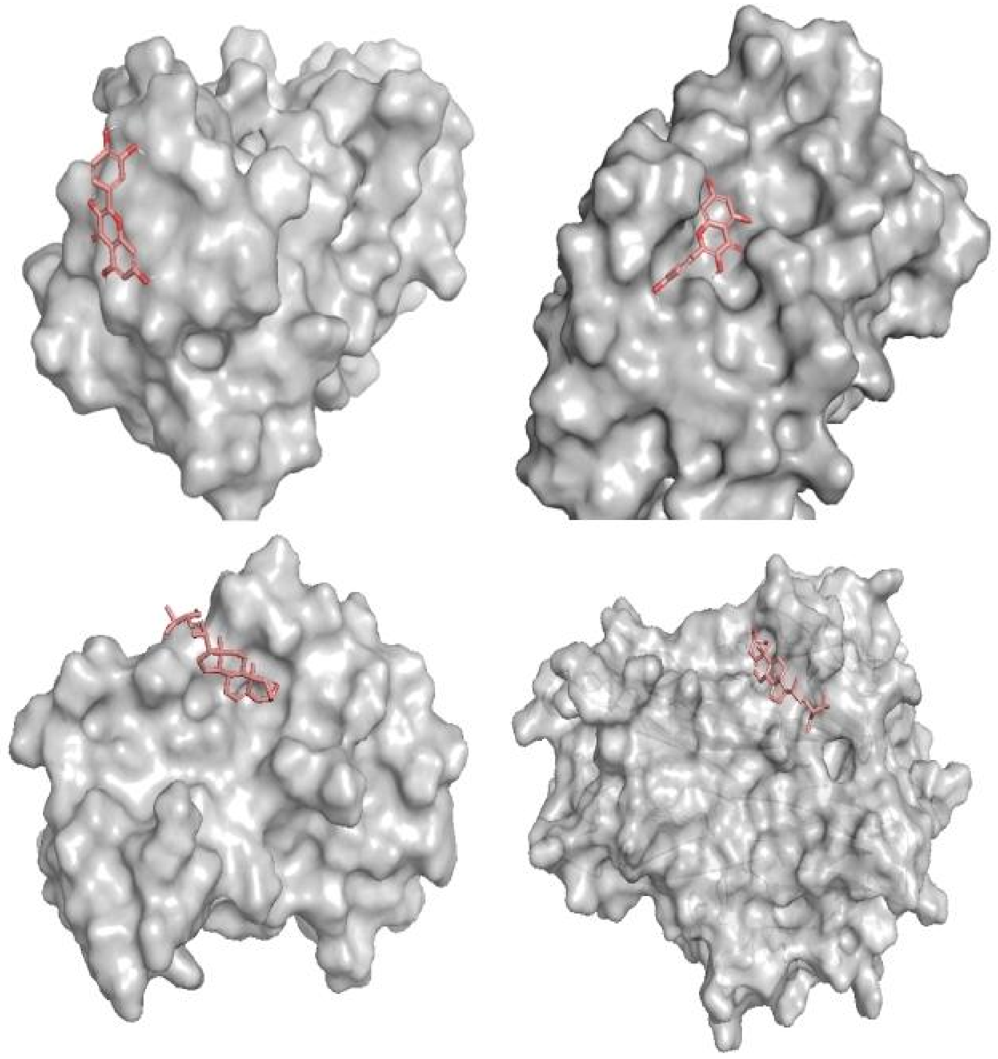

Supplementary Table S1 Acid and bile salt resistance of Lactobacillus isolates

strain	Acid-resistant (pH: 2.5, 3 hours)			Cholestyramine (0.3%, 8 h)		
	0 h (lg CFU/mL)	3 h (lg CFU/mL)	Survival rate(%)	0h (lg CFU/mL)	8h (lg CFU/mL)	Survival rate(%)
Bacillus plantarum	7.78±0.02	7.64±0.05	98.20	8.08±0.03	7.49±0.03	92.7

Supplementary Table S2 Effect of Lactobacillus isolates on growth performance of mice

	Group A	Group B
Initial weight, g	17.9±1.4	17.1±0.4
Final weight, g	31.7±1.9	36.14±2.7
Daily weight gain, mg/day	841.96±402.62	1252.50±205.22
Daily feed intake, g/day/head	3.32	4.4
Feed conversion ratio	3.94	3.51

Supplementary Table S3 Inhibition of GPV by different treatments of mixed decoctions and fermented mixed decoctions (mean ± standard deviation)

	Combination Decoction	Fermented Compound Decoction
Preprocessing Group	0.52±0.0536a	0.51±0.0127a
Combined Treatment Group	0.26±0.0432a	0.31±0.0248a
Post-Processing Group	0.51±0.0711a	0.53±0.0476a

Supplementary Table S4 Top six active ingredients with node connectivity in honeysuckle and gardenia

Molecular Number	Ingredients	Node connectivity
MOL000098	Quercetin	310
MOL000422	Kaempferol	128
MOL000358	β-Sitosterol	78
MOL000449	Stigmasterol	64
MOL003095	5-Hydroxy-7-methoxy-2-(3,4,5-trimethoxyphenyl)chromen-4-one	54
MOL001494	Mandelic acid	8

Supplementary Table S5 Top ten target genes with node connectivity in the PPI network graph of intersecting targets of honeysuckle, gardenia and bovine viral diarrhea

Target Name	Gene Name	Node connectivity
Interleukin-6	IL6	93
Tumor Necrosis Factor	TNF	91
Caspase 3	CASP3	90
Interleukin-1β	IL1B	83
Prostaglandin-Endoperoxide Synthase 2	PTGS2	80
B-cell CLL/lymphoma 2	BCL2	79
Matrix metalloproteinase 9	MMP9	73
Estrogen receptor 1	ESR1	72
Hypoxia-inducible factor 1	HIF1A	71
Transforming growth factor beta-1	TGFB1	70

Supplementary Table S6 Molecular docking validation results

Active ingredients	Target name	Binding energy(kcal/mol)
Quercetin	IL6	-5.06
	TNF	-4.63
	CASP3	-4.12
Shikimic acid	IL6	-6.21
	TNF	-5.11
	CASP3	-4.42
β-sitosterol	IL6	-5.49
	TNF	-7.5
	CASP3	-5.3
Sitosterol	IL6	-6.09
	TNF	-7.26
	CASP3	-5.39
